# Prolonged Dark Chemical Processes in Secondary Organic Aerosols on Filters and in Aqueous Solution

**DOI:** 10.1021/acs.est.4c01647

**Published:** 2024-07-30

**Authors:** Julian Resch, Kangwei Li, Markus Kalberer

**Affiliations:** Department of Environmental Sciences, University of Basel, Klingelbergstrasse 27, 4056 Basel, Switzerland

**Keywords:** SOA composition, dimer formation, esters, chemical analysis, LC-MS

## Abstract

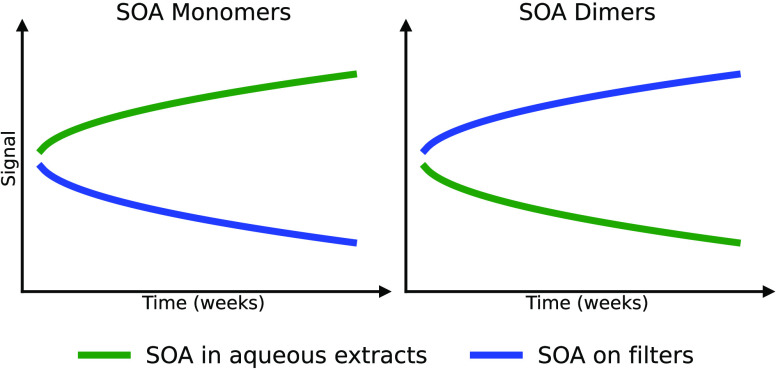

Secondary organic aerosol (SOA) represents a large fraction of atmospheric aerosol particles that significantly affect both the Earth’s climate and human health. Laboratory-generated SOA or ambient particles are routinely collected on filters for a detailed chemical analysis. Such filter sampling is prone to artifactual changes in composition during collection, storage, sample workup, and analysis. In this study, we investigate the chemical composition differences in SOA generated in the laboratory, kept at room temperature as aqueous extracts or on filters, and analyzed in detail after a storage time of a day and up to 4 weeks using liquid chromatography coupled to high-resolution mass spectrometry. We observe significantly different temporal concentration changes for monomers and oligomers in both extracts and on filters. In SOA aqueous extracts, many monomers increase in concentration over time, while many dimers decay at the same time. In contrast, on filters, we observe a strong and persistent concentration increase of many dimers and a decrease of many monomers. This study highlights artifacts arising from SOA chemistry occurring during storage, which should be considered when detailed organic aerosol compositions are studied. The particle-phase reactions on filters can also serve as a model system for atmospheric particle aging processes.

## Abstract 2

Molecular-level understanding of secondary organic aerosol composition and formation mechanisms is essential to reduce the uncertainty regarding environmental and health impacts. This study characterizes particle-phase reactions affecting particle composition during sample storage.

## 1 Introduction

Atmospheric aerosols, especially secondary organic aerosol (SOA), have a large impact on climate and human health.^1−3^ The detailed chemical composition of SOA is highly complex, typically containing thousands of compounds, and a molecular-level understanding of SOA composition and reactivity is important to evaluate sources and to characterize their health and climate effects in detail.^4^ During the past decades, detailed chemical characterization of SOA has become a major research area,^1,5^ and a large number of compounds formed in particle-phase reactions were identified as major components of SOA.^6−8^ Nevertheless, there is still large uncertainty regarding the formation of condensed-phase particle components (such as dimers and higher-order oligomers) to the total mass of SOA, sometimes being reported as high as 75% in freshly nucleated particles.^9−16^

While direct online chemical characterization of aerosol composition (e.g., using aerosol mass spectrometry)^17,18^ has its advantages in fast acquisition time to provide near real-time data, collecting particles onto filters followed by extraction and detailed offline chemical analysis, where particle collection and analysis (e.g., with liquid chromatography coupled to mass spectrometry (LC-MS)) are separated in time, is still the most often used method for both laboratory and ambient studies to characterize and quantify the detailed molecular-level composition of SOA.^5^ A combination of the two is represented by Filter Inlet for Gases and AEROsols (FIGAERO) coupled to detectors such as chemical ionization mass spectrometers (CIMS), where particles are collected on filters for minutes to hours before being evaporated into the gas phase and measured after thermal desorption, which separates the compounds based on their volatility.^19^ These systems have a higher time resolution than LC-MS measurements due to the absence of the filter extraction procedure. Nevertheless, we believe some of the chemical processes happening on the filters as discussed later might still have an effect on compounds collected on filters measured with these systems, especially when the filter collection times are in the time scale of hours or filters have been collected and stored for many days before being analyzed by FIGAERO-CIMS.^20−22^

One fundamental assumption of filter sample analysis is that the particle components are stable during storage and are not significantly affected by filter extraction and other workup procedures. This is likely true for most major inorganic components such as inorganic salts. However, organic compounds may undergo chemical reactions during filter storage or in extracts due to the complex nature of thousands of organic compounds present in aerosol particles. For example, Romonosky et al.^23^ and Wong et al.^24^ investigated the stability of organic aerosol components toward hydrolysis and hydration. The former study suggests that hydrolysis leads to a decomposition of compounds when filter samples are left in water in the dark, while the latter found little change in overall SOA composition during storage in water when focusing their analysis on several major carboxylic acids with direct infusion mass spectrometry for storage timescale of 1–2 days. Wong et al.^24^ further observed changes between a factor of 0.05 and 4 when storing samples on foil substrates exposed to water vapor, which is in a similar range that we observed in the later discussion. Such partially contradictory results reflect the incomplete understanding of the stability of aerosol samples. Other aspects of offline filter processing such as filter extraction and storage of organic aerosol in solvents such as methanol were observed to alter the particle composition leading to methyl ester formation, while no such effects were observed for acetonitrile.^25,26^ More recently, a study from our group^27^ illustrated that different filter storage conditions (i.e., +20 °C (room temperature) versus −20 °C or −80 °C or SOA stored on a filter versus as extracts of a water/acetonitrile mixture) can lead to significantly different overall SOA chemical profiles, especially for samples kept at room temperature.

Here, we explore the chemical composition differences in β-pinene SOA when stored on filters and as extracts at room temperature over days and up to 4 weeks and characterized in detail using ultrahigh-performance liquid chromatography coupled to high-resolution mass spectrometry (UHPLC-HRMS). During aerosol filter sampling, for example, in automated high-volume samplers, filters are often kept at room temperature for many days or several weeks, and thus any compositional changes that might occur over such time scales need to be characterized. We observed significant composition changes in filter extracts as well as in SOA stored on filters, especially persistent concentration increases in many dimers of samples stored on filters. We further performed on-filter “spiking” experiments, where carboxylic acids were nebulized to the filters in excess to induce targeted esterification reactions on filters predeposited with β-pinene SOA, and the results support our observation of increased dimer formation on filters occurring over days. We highlight that these chemical processes after SOA filter collection and extraction are nonnegligible and deserve attention, especially when esters and other oligomers in SOA are characterized from offline particle samples.

## 2 Experimental Section

### 2.1 Filter Sample Collection and Extraction

SOA was generated via O_3_ and OH oxidation of β-pinene (99%, Sigma-Aldrich, Switzerland) with a compact oxidation flow reactor, the “Organic Coating Unit” (OCU),^28^ which produces stable and reproducible SOA mass concentrations. The setup used is given in Figure S1. The average SOA concentrations in the OCU were around 6 mg/m^3^, and around 300 μg SOA per filter quarter were collected (for details see Resch et al.).^27^ Samples were collected on 47 mm PTFE membrane filters with a 0.2 μm pore size (Whatman, Merck, Switzerland). The filters were cut into quarters and placed in 2 mL Eppendorf safe-lock tubes (Eppendorf, Switzerland) under laboratory conditions with approximately 40% RH and 20 °C and either extracted and analyzed immediately or stored in a dark cupboard in the laboratory at +20 °C (room temperature) for approximately 2 days, 1, 2, or 4 weeks before analysis. Five filter samples were collected for each condition (i.e., stored as filter or extract and for different storage times) and injected in duplicates to assess reproducibility of the measurements and results.

The filter extraction procedure was as follows: each filter quarter was placed into 2 mL Eppendorf safe-lock tubes (Eppendorf, Switzerland) and either stored or extracted immediately. 1.5 mL of extraction solvent (1:5 water/acetonitrile (ACN) v/v) was added, and then the samples were vortexed at maximum speed (2400 rpm) for 2 min before being placed on a Fisherbrand Open Air Rocker (Fisher Scientific, Switzerland) for 30 min. The extract was then pipetted into an empty Eppendorf tube to remove the filter before being dried down in a benchtop rotary evaporator (Eppendorf Basic Concentrator Plus; Eppendorf, Switzerland) for 2 h at 45 °C in vacuum concentrator alcohol (V-AL) mode until complete dryness. The samples were then reconstituted with 500 μL of reconstitution solvent (1:10 ACN/water v/v) and vortexed again for 90 s before being split into five aliquots of 100 μL in amber LC-MS vials with 150 μL glass inserts. Acetonitrile shows no evidence of reactions with the analyte molecules.^25^ Samples were then either stored in the dark or placed in the autosampler of the LC instrument for immediate analysis. The extraction procedure was the same for the samples collected from additional “spiking” experiments as described below.

To isolate and accelerate an individual dimerization reaction among the complexity of many possible particle-phase reactions that occur in SOA deposited on filters, we performed “spiking” experiments to see if targeted reactions could be induced on the filters (see Figure S1 for a visualization of the “spiking” process). We generated and collected two identical β-pinene SOA filters under similar conditions as for the experiments described above using the OCU shown in Figure S1 (sampling flow rate: 10 L/min; sampling duration: 240 s; SOA mass concentration: ∼500 μg/m^3^). This results in a β-pinene SOA mass loading of 20 μg per filter. One filter was analyzed without further treatment, while the other filter was “spiked” with carboxylic acids. A solution containing three carboxylic acids (cis-pinic acid, cis-pinonic acid, and pimelic acid, each with a concentration of 0.2 mg/mL in water, all obtained from Sigma-Aldrich, Switzerland) was nebulized and dried with a silica gel dryer, generating dry aerosol particles containing these three carboxylic acids. These carboxylic acid particles were then deposited on the β-pinene SOA particles previously collected on a filter. This spiking process lasted for 70 min with an aerosol flow rate of 2.5 L/min passing through the β-pinene SOA filter, leading to deposition of a total of ca. 12.3 μg carboxylic acids and resulting in an even coating of the carboxylic acid as dry particles onto the β-pinene SOA particles. This procedure not only assures the even distribution of carboxylic acids on the entire filter but also that the spiked carboxylic acids are added to the SOA particles with no or only a minimal amount of liquid water and thus avoids aqueous-phase reaction conditions (as it might occur if the carboxylic acid solutions would be added to the SOA particles through pipetting). The addition of carboxylic acid under these conditions aims to induce targeted particle-phase reactions of the carboxylic acid with alcohols present in SOA to form dimer esters.

### 2.2 UHPLC-HRMS Analysis

UHPLC-HRMS analysis of filter extracts was performed using a Thermo Vanquish Horizon UHPLC with binary pump and split sampler (Thermo Fisher Scientific, Reinach, Switzerland), equipped with a Waters HSS t3 UPLC column (100 mm × 2.1 mm, 1.8 μm, Waters AG, Baden, Switzerland), connected to an Orbitrap Q Exactive Plus (Thermo Fisher Scientific, Switzerland), which was used in negative polarity electrospray mode (all reported compounds are assumed to be the singly charged [M–H]^−^ species). The scan parameters were set to full MS, a scan range of *m*/*z* 85–1000, an automated gain control target of 3 × 10^6^, and a resolution of 70 000 with a maximum injection time of 25 ms. The mobile phases, where all solvents were Optima LC-MS grade, were obtained from Fisher Scientific (Switzerland). Water +10 mM acetic acid (mobile phase A) and methanol (mobile phase B) were run at a flow rate of 400 μL/min in a 30 min method at the following gradient: 99.9% A from 0 to 2 min, a linear ramp up to 99.9% B from 2 to 26 min, 99.9% B was held until 28 min, and then switching to 99.9% A for column reequilibration from 28.1 to 30 min. To monitor system stability of the LC-MS over the course of measurements, daily calibrations were done using the Thermo Scientific Pierce Negative Ion Calibration Solution (Fisher scientific, Switzerland) along with injections of a HPLC gradient test mix (Sigma-Aldrich, Switzerland) and calibration curves in the range of 10 ng/mL to 10 μg/mL of a standards mixture of *cis*-pinonic acid, camphoric acid, 4-hydroxybenzoic acid, 1,2-naphthoquinone and pimelic acid (all obtained from Sigma-Aldrich, Merck, Switzerland). Filter blanks and solvent blanks were injected after every three sample injections to monitor the background intensity of the system and to check for carryover.

For the “spiking” experiments, the only difference in analysis was the use of an ACQUITY UPLC I-Class PLUS System with a Binary Solvent Manager (BSM) and a Sample Manager with a Flow-Through Needle (SM-FTN) (Waters AG, Switzerland) in front of the Orbitrap MS. Using the two LC instruments resulted in a slight retention time shift (which was accounted for using an HPLC Gradient System Diagnostics Mix from Sigma-Aldrich (Merck, Switzerland)). Additionally, the flow rate was reduced to 300 μL/min due to higher backpressure in this system. The mobile phases and gradients remained the same. Untargeted LC-MS data analysis was performed in R 4.2.1 (R Core Team, Austria) in RStudio 2022.07.01 (Boston, Massachusetts) using the XCMS package for untargeted peak detection.^29−31^

**Figure 1:**
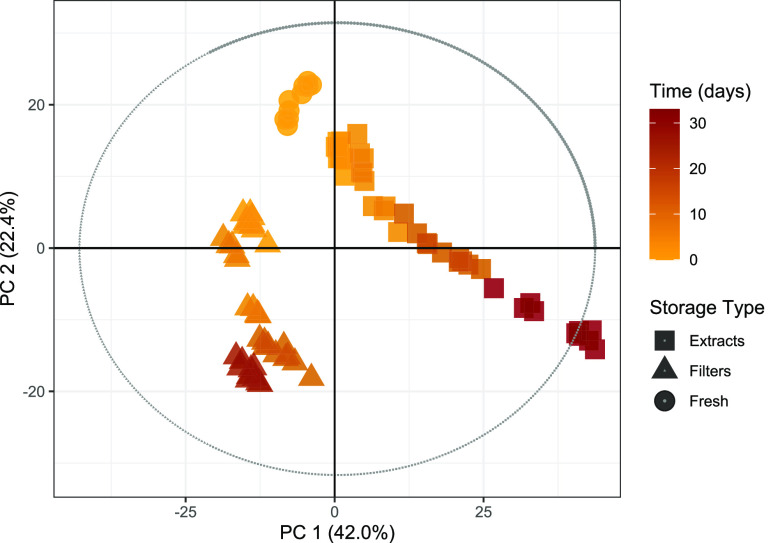
Log_10_(*x*) normalized PCA score plot of 4735 detected peaks in all samples. Shapes represent the storage type, and the colorbar represents the time between collection and analysis in days. The proportion of variance is displayed as PC 1 and 2, as well as *R*^2^*X*[1] = 0.420 and *R*^2^*X*[2] = 0.224. The predictive power of the model is given as *Q*^2^[1] = 0.405 and *Q*^2^[2] = 0.370.

In order to observe trends and variation in the data set, principal component analysis was used on the untargeted peaks identified. Multivariate statistical analysis was performed with SIMCA 17 (Sartorius, Germany); model performance was evaluated using *R*^2^ values as a measure of the proportion of variance explained by the model. The *Q*^2^ value estimates the predictive power of the model through 7-fold cross-validation using randomly selected test and train subsets taken from the data set. Hotelling’s *T*^2^ statistic was used to identify potential outliers in the data set. Hotelling’s *T*^2^ ellipse (95%) is represented by the gray dotted line in Figure 1. More details are given by Resch et al.^27^

## 3 Results and Discussion

The main focus of this study is to explore the temporal artifactual changes of β-pinene SOA composition that might occur during storage of sample extracts and filter samples at room temperature over days and up to several weeks, focusing specifically on dimer formation or decomposition. β-pinene was chosen as a representative biogenic SOA.^1^ β-pinene is one of the main biogenic VOCs^32−34^ and has been used for many previous laboratory SOA studies.^16,35,36^ The compositional changes occurring on filters during storage over days also mimic particle-phase processes that might occur during the lifetime of SOA particles in the atmosphere.

### 3.1 Overall Characteristics

Figure 1 shows a principal component analysis (PCA) score plot for log_10_(*x*) normalized peak intensities of 4735 peaks for all samples analyzed in this study and illustrates the significant differences in the temporal behavior of chemical composition between the SOA samples stored on filters (triangles) and as extracts (squares). The extract composition shows stronger changes over time in both principal component (PC) 1 and PC 2 compared to the samples stored on filters. The large distance between the composition of the directly extracted and analyzed samples (circles) and the samples stored for 2–3 days demonstrates that the most significant changes in particle composition occur over the first 2–3 days. After this initial change, the filter samples show less variation over time, but for extracts, a continuous and strong change in composition is observed at least up to 4–5 weeks.

**Figure 2:**
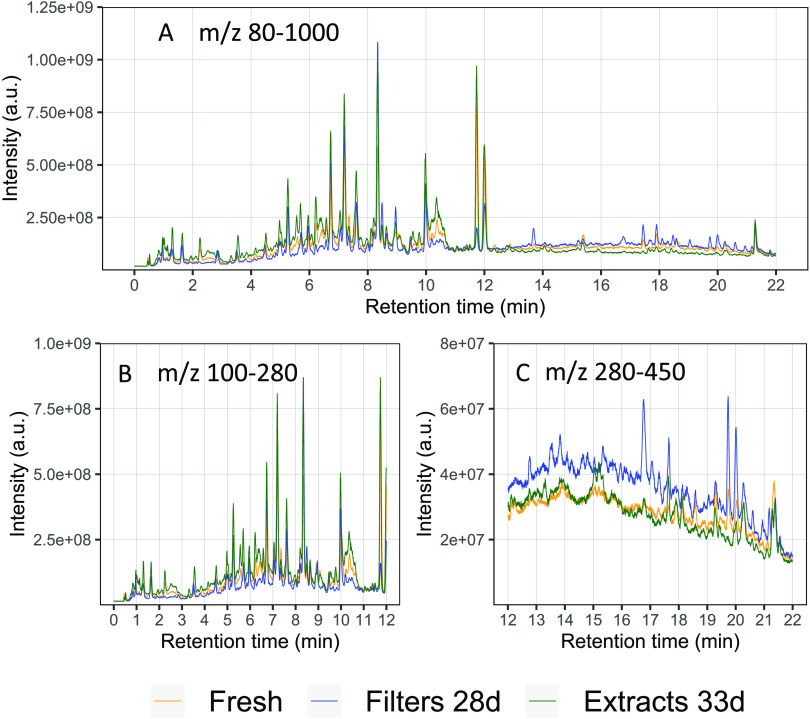
(A) TIC representing fresh SOA extracts and SOA stored on a filter and as extract for 28 and 33 days, respectively. (B) TIC of the monomer region with *m*/*z* 100–280 and retention times between 0 and 12 min. (C) TIC of the dimer region with *m*/*z* 280–450 between 12 and 22 min. The stored filter and extract samples show inverse temporal effects in the monomer and dimer region.

One explanation for this observed difference between samples stored as extracts or on filters could be the hydrolysis of compounds, such as esters, decomposing into their monomeric components. It has been shown that esters form a large fraction of oligomers.^7,8^ Additionally, reactions of stabilized Criegee intermediates (SCI) with carboxylic acids have been shown to form dimer compounds containing hydroperoxide functional groups, which are known to decay quickly when stored in aqueous solutions.^37^ These significant changes of particle composition over time, especially in SOA extracts, are also seen in the total ion chromatogram (TIC) shown in Figure 2A, including a fresh sample (immediately extracted and analyzed after collection), an extract stored for 33 days, and a filter stored for 28 days.

Monomers (*m*/*z* 100–280) and dimers (*m*/*z* 280–450)^38^ generally elute at different retention times^35^ and thus the chromatogram can be separated into two regions (Figure 2B,C). In the earlier eluting monomer region (Figure 2B), we observe an increase in the overall signal intensity for the stored extracts (green) compared to that of the fresh samples (yellow). The filters (blue) on the other hand show an overall decreased signal in the chromatogram. The retention time range when most oligomers elute (>12 min, Figure 2C) shows the opposite trend: for samples stored as extracts, the signal intensities stay relatively constant over 4 weeks or decrease slightly (especially peaks after 18 min retention time), while the signal intensity for samples stored on filters increases significantly over time. The same results are given as a base peak chromatogram (BPC) in Figure S2.

These observations suggest that on filters oligomers are formed through particle-phase reactions even under conditions where no photochemistry occurs and in the absence of oxidants, continuously changing the composition of SOA particles and leading to a decrease in monomer concentrations. These reactions observed on filters during storage at room temperature are similar to particle composition changes seen for aging of SOA in other studies, where oligomer formation in the particle phase has been reported for time scales of minutes to hours.^39−42^ An additional effect that could explain the temporal changes seen on filters for monomers could be the depletion of higher-volatility species through evaporative losses.^43−47^ While this will certainly have an effect in the reduction of monomers over time, it would likely mainly explain part of the signal decrease we observe for monomers during the first day (as continuous evaporation over weeks is unlikely), and it would not explain the continuous concentration increase of dimers on filters due to their high molecular weight and thus low volatility. Hence, we assume that it is a combination of several effects taking place during the storage of filters.

Note that even though a majority of the changes observed are most prominent in room-temperature samples, there are still changes in the aerosol composition at storage temperatures at and below −20 °C. As discussed in our previous study,^27^ storage temperatures of −20 °C and −80 °C significantly reduce these changes, but some chemical composition changes still occur.

### 3.2 Individual Compounds

We also characterized the temporal behavior of several individual compounds that have previously been tentatively identified in the literature as dimer esters in β-pinene SOA samples.^7,8,35,36,48,49^ A detailed list of the compounds (*n* = 33) investigated is given in Table S1. From this list of previously identified ester dimers, we selected the 10 highest intensity peaks in the fresh samples and the 12 highest intensity peaks in the 4-week-old filter samples, resulting in 18 chromatographic peaks investigated in detail, which are given in Table S2. These selection criteria avoid biased observations toward the previously discussed formation/decomposition processes in extracts/filters and enable a broad coverage of dimer esters. Extracted ion chromatograms and time series plots are given for each of the 18 *m*/*z* analyzed (see Figures S3–S20). The individual compounds in our analysis show a wide range of temporal behaviors, depending on the specific reactions involved. All 18 *m*/*z* are identical to esters previously identified in the literature, but all *m*/*z* show several isomers in their extracted ion chromatogram (EIC) and there are likely numerous isomers without an ester functional group. A prominent trend among almost all EICs is the increase of several isomeric peaks on the filter samples stored over time.

**Figure 3:**
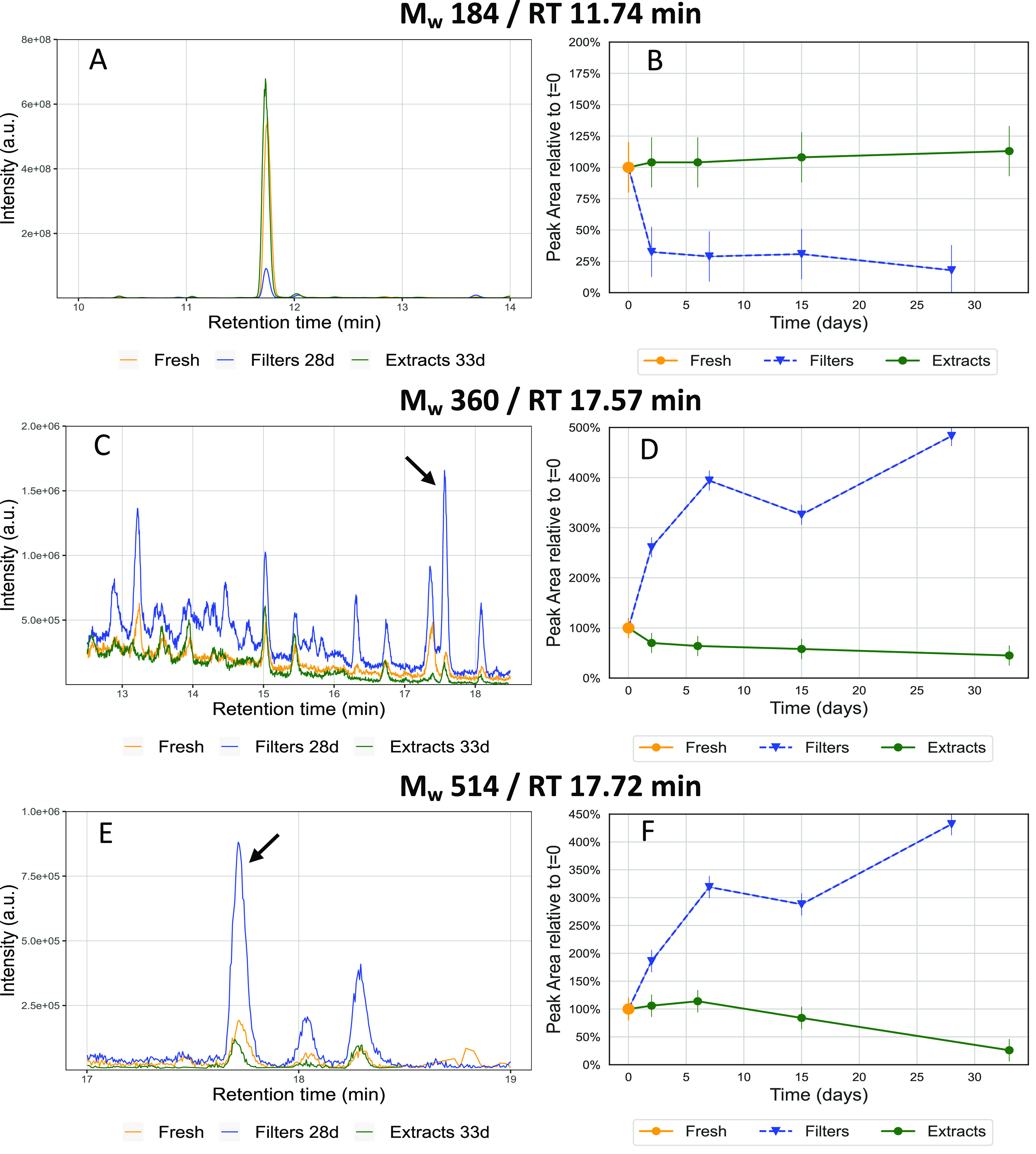
EICs and time series plots of fresh and stored filters and extracts of (A, B) *M*_W_ 184 (*m*/*z* 183.1027) monomer, which shows a significant decrease after the first day when stored on filters and a slight increase in signal intensity when stored as extracts; (C, D) *M*_W_ 360 (*m*/*z* 359.1706) dimer, which shows a strong continuous increase in signal intensity when stored on filters and a decrease in extracts. Additionally, several new isomers are detectable in the EIC of the stored filter samples. (E, F) *M*_W_ 514 (*m*/*z* 513.1954) trimer, which shows a temporal behavior similar to that of the dimer. Error bars represent the total relative uncertainty of ±20% as described in Resch et al.

In Figure 3, we show the temporal behavior of representative examples of compounds in the monomer, dimer, and trimer mass range, respectively. Cis-pinonic acid (*M*_W_ 184, a monomer at *m*/*z* 183.1027, C_10_H_16_O_3_, RT 11.73 min) is the peak with the highest intensity in fresh samples and shows a significant decrease by almost 75% in signal intensity in the 3 days after collection when samples are stored on filters and stays relatively stable in the following 3–4 weeks (this effect is even seen when the filters are stored at −20 °C or −80 °C).^27^ In contrast, the signal intensity of the extracts seems to be stable within the measurement uncertainty over the course of a month. The significant decrease of cis-pinonic acid on filter samples could possibly be explained by the formation of oligomer compounds in the SOA formed through condensed-phase reactions^41^ as well as through desorptive losses as previously observed by Glasius et al.^50^

An EIC of the previously identified *M*_W_ 360 (*m*/*z* 359.1706, C_17_H_28_O_8_, RT 17.57 min)^8^ dimer is displayed in Figure 3C. While the fresh samples show around eight distinguishable isomer peaks eluting between 13 and 18 min, the stored filter samples exhibit more than twice as many (at least 17 distinguishable isomer peaks). Several of these isomers are not detectable in the fresh or stored extract samples. The strongest increase in signal intensity is observed for the peak at 17.57 min, which shows an increase of around 500% over 4 weeks in comparison to the fresh samples (Figure 3D). Possible explanations for the increase of dimers on filters are particle-phase esterification reactions of an alcohol and a carboxylic acid or through Baeyer–Villiger reactions between ketones and organic peroxides.^41^

We additionally searched for trimers (C_20–30_) in our samples and found several candidates that we could annotate with an according chemical formula using the XCalibur (Thermo Fisher Scientific, Switzerland) software. Figure 3E displays the EIC of a trimer *M*_W_ 514 (*m*/*z* 513.1954, C_24_H_34_O_12_, RT 17.72 min), which shows a strong signal intensity increase in the stored filter samples in comparison to the fresh samples for multiple isomers. The extracts show the opposite trend, with a significant decrease in peak height over time (Figure 3F). This temporal behavior could indicate that some trimers listed in this work might also include an ester group, although this could not be confirmed and the structural identification of these trimers was not the focus of this study. Several other trimers are presented in Figures S21–S25.

**Figure 4:**
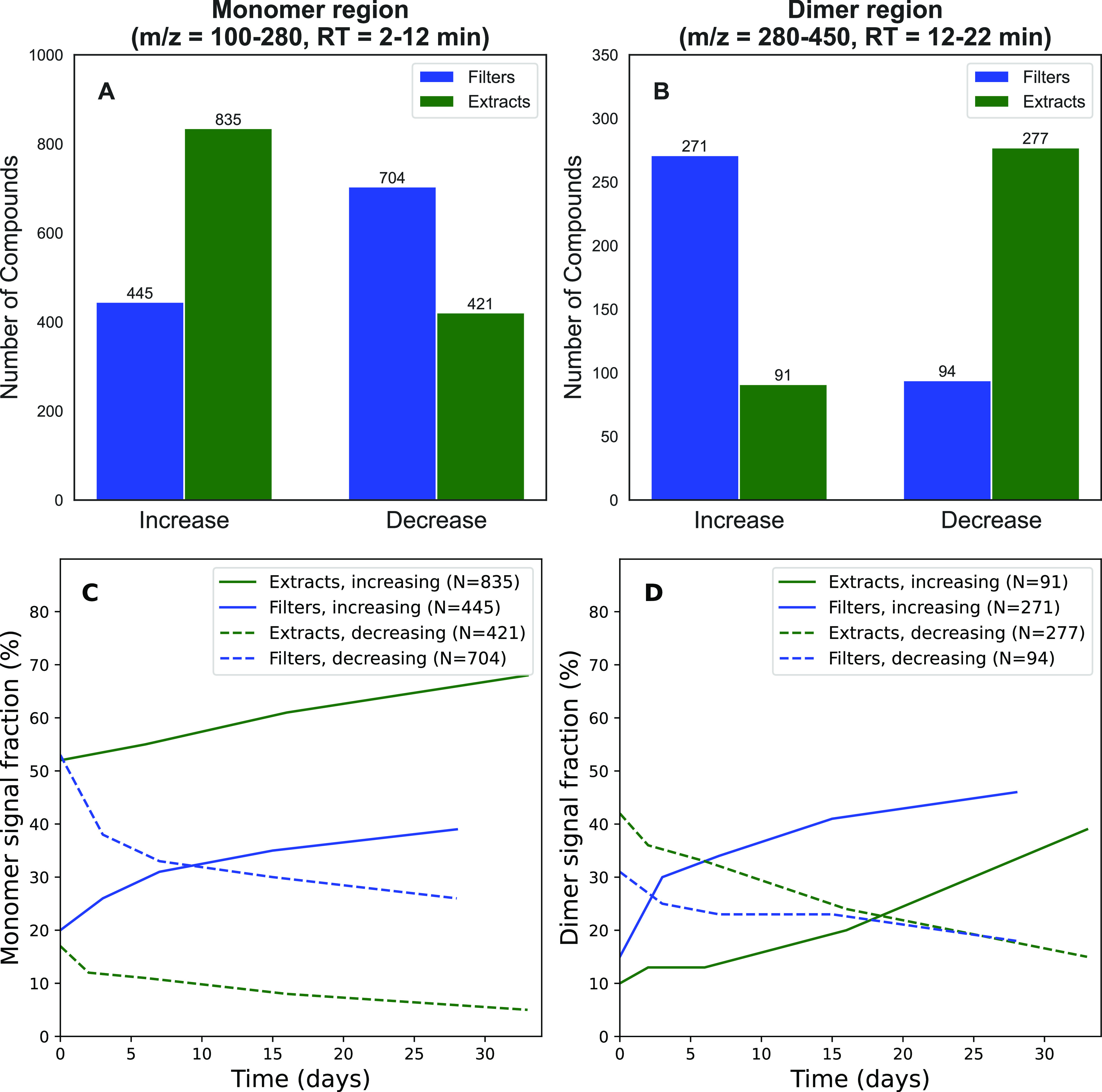
Overall number of compounds in the (A) monomer region and (B) dimer region that show an increasing or decreasing trend over 4 weeks when stored as extracts (green) or on filters (blue). The signal fraction of these categorized compounds compared to the total signal intensity of all monomers or dimers is given in panels (C, D), respectively. The remaining signal fraction shows no clear temporal trends and is not displayed here.

The selected *m*/*z* discussed in Figures 3 and S3–S25 are only a few of the thousands of compounds present in these SOA samples. Therefore, we further examined the temporal trends of all detected peaks in the monomer and dimer regions to determine the total amount of increasing and decreasing compounds (see Figure 4). Two statistical selection criteria were applied in order to categorize compounds with an increasing or decreasing trend: (a) for each of the five points analyzed (i.e., fresh, 2 days, 1 week, 2 weeks, and 4 weeks stored), the subsequent point in time must be higher (for increasing) or lower (for decreasing) than the previous one, and this condition must be true for *n* ≥ 3 points with a maximum of *n* = 4. (b) a linear fit through the points must have a positive (for increasing) or negative (for decreasing) slope. Both conditions (a) and (b) must be fulfilled. 1514 peaks in the filter samples met these criteria for a clear temporal trend (1149 monomers and 365 dimers) and 1624 peaks in extract samples (1256 monomers and 368 dimers). In Figure 4A, representing the monomers, almost twice as many peaks increase in the extracts over time compared with the number of decreasing peaks. Figure 4B summarizes the temporal trend of dimeric compounds, illustrating that on filters almost three times more dimers increase over time than decrease in concentration. Figure 4C,D shows the signal intensity fraction of all compounds that have increasing and decreasing temporal trends (relative to the sum of all monomers or dimers) on filters and extracts in the monomer and dimer regions, respectively. The number of peaks included in Figure 4C,D is the same as displayed in panels A and B. These signal intensity fractions can be interpreted as a proxy of mass fractions. A large fraction (65–75% in the monomer and 45–65% in the dimer region) of the total signal intensity shows increasing and decreasing trends, suggesting that a large part of compounds in SOA shows such temporal changes.

These results highlight that these temporal effects illustrated in Figure 3, and especially the persistent growth of dimers on filters, are observed for hundreds of compounds in complex SOA samples. Additionally, this overall trend reinforces the hypothesis that such temporal behaviors could be explained by a general mechanism, e.g., hydrolysis leading to decomposition of dimers and formation of monomers in SOA aqueous extracts, as also observed by Witkowski et al.^51^ In contrast, on filters, continuous SOA processing occurs and therefore results in a removal of monomers and formation of dimers. We acknowledge that with the high SOA concentrations used in this study, some constituents may have partitioned more from the gas phase into the particle phase compared to atmospheric conditions. We still believe the observed composition changes on filters are not dominated by such concentration effects because we observe similar effects when the SOA concentrations in the OCU are reduced by an order of magnitude as in the “spiking” experiments discussed in the next section.

### 3.3 Accelerating Particle-Phase Dimer Formation in β-Pinene SOA

In order to further test our hypothesis of continuous organic particle-phase reactions on filters over days, we nebulized a large excess of three carboxylic acid standards, pinic acid, pimelic acid, and *cis*-pinonic acid, onto filters (in order to evenly distribute dry carboxylic acid aerosol particles) preloaded with β-pinene SOA, which is expected to induce some targeted esterification reactions onto filters. We refer to this process as “spiking” (see Experimental Section and Figure S1) and compared their compositional evolution over time with β-pinene SOA filters without this addition, acting as controls, referred to as “filter-only”. We monitored the formation of a previously described dimer ester (*M*_W_ 358) from the esterification reaction of pinic acid (*M*_W_ 186, *m*/*z* 185.0819, C_9_H_14_O_4_) and diaterpenylic acid (*M*_W_ 190, *m*/*z* 189.0768, C_8_H_14_O_5_).^52^ As diaterpenylic acid was previously identified as a major monomeric component in dimer formation processes through esterification.^48,53,54^ we additionally investigated possible dimers being formed on the filters through reaction of diaterpenylic acid with the other two deposited carboxylic acids such as pimelic acid and *cis*-pinonic acid.

**Figure 5:**
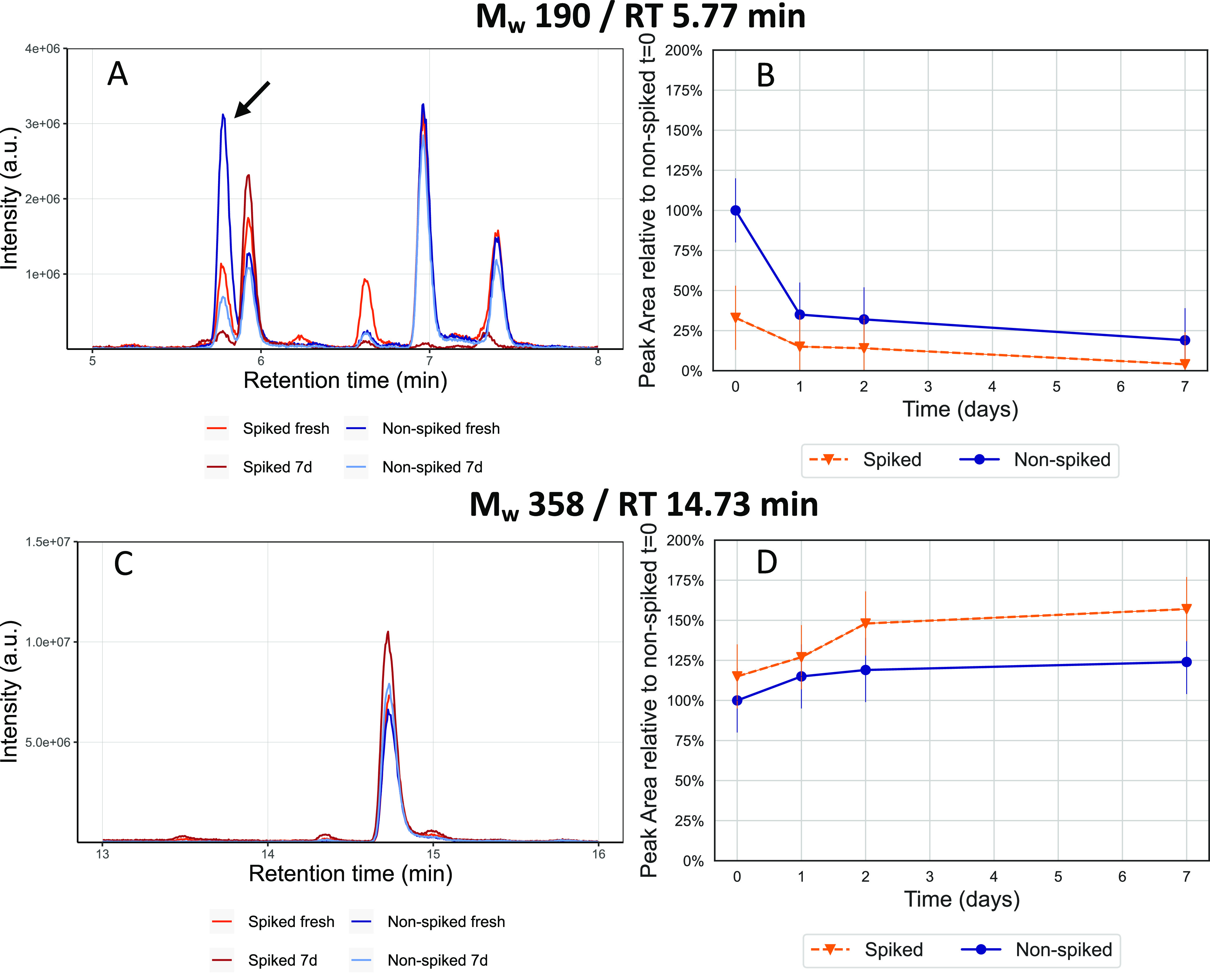
(A, B) EIC and time series plot of the “filter+spiking” and “filter-only” fresh and 7-day old filter samples for diaterpenylic acid (*M*_W_ 190, *m*/*z* 189.0776). A clear decrease in signal intensity is observed over time for both the “filter+spiking” and “filter-only” samples, suggesting particle-phase reaction of diaterpenylic acid over days in the SOA samples, although a much stronger decrease is observed within hours for the “filter+spiking” samples. The signal intensities in panels (B, D) are normalized to the fresh “filter-only” samples. (C, D) EIC and time series plot of the “filter + spiking” and “filter-only” fresh and 7-day old filter samples for the *M*_W_ 358 (*m*/*z* 357.1550) assigned to a dimer ester of pinic acid and diaterpenylic acid. There is a stronger increase in the “filter + spiking” samples over time compared to the “filter-only” samples, indicating that an excess of pinic acid in the spiked samples promotes the dimer ester formation.

Figure 5 shows the EICs and the temporal behavior of both diaterpenylic acid (*M*_W_ 190, panel A and B) and the *M*_W_ 358 ester (panels C and D) for the “filter+spiking” and “filter-only” samples over a week. Through comparison of MS/MS measurements with the literature^52^ (see Figure S26), we tentatively assigned the peak eluting at 5.77 min (marked with an arrow in Figure 5A) as diaterpenylic acid. The signal for both sample types is normalized to the fresh “filter-only”, as it can be assumed that the initial concentration of diaterpenylic acid deposited onto the filters and the β-pinene SOA is the same for both “filter+spiking” and “filter-only”. The signal intensity in both the “filter+spiking” and “filter-only” samples decreases by more than a half during the first day of storage. The fresh signal for the “filter+spiking” samples is significantly lower (at about 30%) than the “filter-only” signal, which can be explained by the high concentration of reaction partners (i.e., the three carboxylic acids) on the “filter+spiking” samples with which diaterpenylic acid reacts in the 2–3 h between spiking and prior to analysis.

We also observed a strong decrease in the two isomers of diaterpenylic acid eluting at 6.96 and 7.39 min for the “filter + spiking” samples (only a time series of the RT 6.96 min peak is presented, Figure S27), while the “filter-only” samples remain stable over a week. It is likely these isomers are structurally similar and might therefore also react with different carboxylic acids to form oligomers, illustrating again the complexity of particle-phase reactions in SOA.

The M_W_ 358 dimer ester shows a stronger increase over time in the “filter + spiking” compared to the “filter-only” samples (Figure 5C,D). This enhanced formation of the dimer in combination with the observed decrease in the precursor over time on the treated filters provides further evidence that dimers are continuously formed in SOA over many days. As control experiments, we nebulized the three carboxylic acids onto filters in the absence of β-pinene SOA and no dimer formation was observed (see Figures S27–S30), suggesting that the observed dimer is indeed a reaction product of the nebulized acid and a SOA component. *M*_W_ 332 and 356 dimers, corresponding to diaterpenylic acid and pimelic or *cis*-pinonic acid dimer esters are given in Figures S28 and S29, both of which also show a stronger increase in signal intensity over time on the “filter + spiking” samples. Note that the pathways presented here are only one of many complex and often still poorly understood dimer formation pathways in secondary organic aerosol,^8,35,52,55−59^ such as the recently described formation of the *M*_W_ 358 dimer ester through particle-phase reactions of alcohols with acylperoxyhemiacetal by Kenseth et al.,^60^ similar to oligomer formation reactions reported by Claflin et al.^41^

### 3.4 Atmospheric Implications

The results of this study show significant changes of the SOA molecular composition over several weeks after particle collection, when laboratory-generated β-pinene SOA particles are stored either on filters or as extracts in aqueous solution. We suggest two dominant processes explaining this observation: (a) continuous particle-phase chemical reactions on filters and (b) hydrolysis of dimers and higher oligomers in aqueous solution. In particular, we propose SOA condensed-phase reactions occurring on filters as important but overlooked SOA processes in previous studies, which lead to the formation of dimers and thus alter particle dimer composition compared to the time of sampling. As illustrated in Figure 3C, not only relative concentration changes of SOA components are observed over time but also new compounds are formed for some *m*/*z*. The total number of peaks in the samples, however, stays constant within 5%.

Our study raises concerns for filter-based offline chemical analyses, especially for detailed molecular-level organic analyses, where such stability issues need to be considered. We demonstrated in a recent study that storing samples at −20 °C or below can significantly reduce compositional changes over time although not completely prevent these changes.^27^ This strongly suggests that filter samples should be immediately stored in a freezer and not kept at room temperature over days before the analysis of organic components.

Continuous reactions of SOA components over days and weeks on filters might also mimic dark aging particle-phase processes of SOA in particles with low water content in the ambient atmosphere, causing an increase in dimer formation and compositional complexity over the entire lifetime of SOA particles in the atmosphere. Such long processing times are usually not accessible with existing experimental methods, e.g., atmospheric simulation chamber or flow tube studies. While the findings presented in this study focus on dimer esters, the processes described above are likely not limited to this compound class but might affect the overall chemical composition of SOA.

## Supporting Information Available

Tables including the compounds discussed in this study; as well as EICs and time series plots of those compounds; and additional illustration of the experimental setup and methods used (PDF)

## References

[ref1] Hallquist M.; Wenger J. C.; Baltensperger U.; Rudich Y.; Simpson D.; Claeys M.; Dommen J.; Donahue N. M.; George C.; Goldstein A. H.; Hamilton J. F.; Herrmann H.; Hoffmann T.; Iinuma Y.; Jang M.; Jenkin M. E.; Jimenez J. L.; Kiendler-Scharr A.; Maenhaut W.; McFiggans G.; Mentel T. F.; Monod A.; Prévôt A. S. H.; Seinfeld J. H.; Surratt J. D.; Szmigielski R.; Wildt J. The Formation, Properties and Impact of Secondary Organic Aerosol: Current and Emerging Issues. Atmos. Chem. Phys. 2009, 9 (14), 5155–5236. 10.5194/acp-9-5155-2009.

[ref2] Jimenez J. L.; Canagaratna M. R.; Donahue N. M.; Prevot A. S. H.; Zhang Q.; Kroll J. H.; DeCarlo P. F.; Allan J. D.; Coe H.; Ng N. L.; Aiken A. C.; Docherty K. S.; Ulbrich I. M.; Grieshop A. P.; Robinson A. L.; Duplissy J.; Smith J. D.; Wilson K. R.; Lanz V. A.; Hueglin C.; Sun Y. L.; Tian J.; Laaksonen A.; Raatikainen T.; Rautiainen J.; Vaattovaara P.; Ehn M.; Kulmala M.; Tomlinson J. M.; Collins D. R.; Cubison M. J.; Dunlea E. J.; Huffman J. A.; Onasch T. B.; Alfarra M. R.; Williams P. I.; Bower K.; Kondo Y.; Schneider J.; Drewnick F.; Borrmann S.; Weimer S.; Demerjian K.; Salcedo D.; Cottrell L.; Griffin R.; Takami A.; Miyoshi T.; Hatakeyama S.; Shimono A.; Sun J. Y.; Zhang Y. M.; Dzepina K.; Kimmel J. R.; Sueper D.; Jayne J. T.; Herndon S. C.; Trimborn A. M.; Williams L. R.; Wood E. C.; Middlebrook A. M.; Kolb C. E.; Baltensperger U.; Worsnop D. R. Evolution of Organic Aerosols in the Atmosphere. Science 2009, 326 (5959), 1525–1529. 10.1126/science.1180353. 20007897

[ref3] Pöschl U.; Shiraiwa M. Multiphase Chemistry at the Atmosphere-Biosphere Interface Influencing Climate and Public Health in the Anthropocene. Chem. Rev. 2015, 115 (10), 4440–4475. 10.1021/cr500487s. 25856774

[ref4] Johnston M. V.; Kerecman D. E. Molecular Characterization of Atmospheric Organic Aerosol by Mass Spectrometry. Annu. Rev. Anal. Chem. 2019, 12, 247–274. 10.1146/annurev-anchem-061516-045135. 30901261

[ref5] Nozière B.; Kalberer M.; Claeys M.; Allan J.; D’Anna B.; Decesari S.; Finessi E.; Glasius M.; Grgić I.; Hamilton J. F.; Hoffmann T.; Iinuma Y.; Jaoui M.; Kahnt A.; Kampf C. J.; Kourtchev I.; Maenhaut W.; Marsden N.; Saarikoski S.; Schnelle-Kreis J.; Surratt J. D.; Szidat S.; Szmigielski R.; Wisthaler A. The Molecular Identification of Organic Compounds in the Atmosphere: State of the Art and Challenges. Chem. Rev. 2015, 115 (10), 3919–3983. 10.1021/cr5003485. 25647604

[ref6] Tolocka M. P.; Jang M.; Ginter J. M.; Cox F. J.; Kamens R. M.; Johnston M. V. Formation of Oligomers in Secondary Organic Aerosol. Environ. Sci. Technol. 2004, 38 (5), 1428–1434. 10.1021/es035030r. 15046344

[ref7] Müller L.; Reinnig M. C.; Warnke J.; Hoffmann T. Unambiguous Identification of Esters as Oligomers in Secondary Organic Aerosol Formed from Cyclohexene and Cyclohexene/α-Pinene Ozonolysis. Atmos. Chem. Phys. 2008, 8 (5), 1423–1433. 10.5194/acp-8-1423-2008.

[ref8] Kristensen K.; Watne ÅK.; Hammes J.; Lutz A.; Petäjä T.; Hallquist M.; Bilde M.; Glasius M. High-Molecular Weight Dimer Esters Are Major Products in Aerosols from α-Pinene Ozonolysis and the Boreal Forest. Environ. Sci. Technol. Lett. 2016, 3 (8), 280–285. 10.1021/acs.estlett.6b00152.

[ref9] Bellcross A.; Bé A. G.; Geiger F. M.; Thomson R. J. Molecular Chirality and Cloud Activation Potentials of Dimeric α-Pinene Oxidation Products. J. Am. Chem. Soc. 2021, 143 (40), 16653–16662. 10.1021/jacs.1c07509. 34605643

[ref10] Couvidat F.; Vivanco M. G.; Bessagnet B. Simulating Secondary Organic Aerosol from Anthropogenic and Biogenic Precursors: Comparison to Outdoor Chamber Experiments, Effect of Oligomerization on SOA Formation and Reactive Uptake of Aldehydes. Atmos. Chem. Phys. 2018, 18 (21), 15743–15766. 10.5194/acp-18-15743-2018.

[ref11] Ehn M.; Thornton J. A.; Kleist E.; Sipilä M.; Junninen H.; Pullinen I.; Springer M.; Rubach F.; Tillmann R.; Lee B.; Lopez-Hilfiker F.; Andres S.; Acir I. H.; Rissanen M.; Jokinen T.; Schobesberger S.; Kangasluoma J.; Kontkanen J.; Nieminen T.; Kurtén T.; Nielsen L. B.; Jørgensen S.; Kjaergaard H. G.; Canagaratna M.; Maso M. D.; Berndt T.; Petäjä T.; Wahner A.; Kerminen V. M.; Kulmala M.; Worsnop D. R.; Wildt J.; Mentel T. F. A Large Source of Low-Volatility Secondary Organic Aerosol. Nature 2014, 506 (7489), 476–479. 10.1038/nature13032. 24572423

[ref12] Hall W. A. IV; Johnston M. V. Oligomer Content of α-Pinene Secondary Organic Aerosol. Aerosol Sci. Technol. 2011, 45 (1), 37–45. 10.1080/02786826.2010.517580.

[ref13] Gao Y.; Hall W. A.; Johnston M. V. Molecular Composition of Monoterpene Secondary Organic Aerosol at Low Mass Loading. Environ. Sci. Technol. 2010, 44 (20), 7897–7902. 10.1021/es101861k. 20853884

[ref14] Kourtchev I.; Fuller S. J.; Giorio C.; Healy R. M.; Wilson E.; O’Connor I.; Wenger J. C.; McLeod M.; Aalto J.; Ruuskanen T. M.; Maenhaut W.; Jones R.; Venables D. S.; Sodeau J. R.; Kulmala M.; Kalberer M. Molecular Composition of Biogenic Secondary Organic Aerosols Using Ultrahigh-Resolution Mass Spectrometry: Comparing Laboratory and Field Studies. Atmos. Chem. Phys. 2014, 14 (4), 2155–2167. 10.5194/acp-14-2155-2014.

[ref15] Kalberer M.; Paulsen D.; Sax M.; Steinbacher M.; Dommen J.; Prevot A. S. H.; Fisseha R.; Weingartner E.; Frankevich V.; Zenobi R.; Baltensperger U. Identification of Polymers as Major Components of Atmospheric Organic Aerosols. Science 2004, 303 (5664), 1659–1662. 10.1126/science.1092185. 15016998

[ref16] Kenseth C. M.; Hafeman N. J.; Huang Y.; Dalleska N. F.; Stoltz B. M.; Seinfeld J. H. Synthesis of Carboxylic Acid and Dimer Ester Surrogates to Constrain the Abundance and Distribution of Molecular Products in α-Pinene and β-Pinene Secondary Organic Aerosol. Environ. Sci. Technol. 2020, 54 (20), 12829–12839. 10.1021/acs.est.0c01566. 32813970

[ref17] Jayne J. T.; Leard D. C.; Zhang X.; Davidovits P.; Smith K. A.; Kolb C. E.; Worsnop D. R. Development of an Aerosol Mass Spectrometer for Size and Composition Analysis of Submicron Particles. Aerosol Sci. Technol. 2000, 33 (1–2), 49–70. 10.1080/027868200410840.

[ref18] Nash D. G.; Baer T.; Johnston M. V. Aerosol Mass Spectrometry: An Introductory Review. Int. J. Mass Spectrom. 2006, 258 (1–3), 2–12. 10.1016/j.ijms.2006.09.017.

[ref19] Lopez-Hilfiker F. D.; Mohr C.; Ehn M.; Rubach F.; Kleist E.; Wildt J.; Mentel T. F.; Lutz A.; Hallquist M.; Worsnop D.; Thornton J. A. A Novel Method for Online Analysis of Gas and Particle Composition: Description and Evaluation of a Filter Inlet for Gases and AEROsols (FIGAERO). Atmos. Meas. Tech. 2014, 7 (4), 983–1001. 10.5194/amt-7-983-2014.

[ref20] Cai J.; Daellenbach K. R.; Wu C.; Zheng Y.; Zheng F.; Du W.; Haslett S. L.; Chen Q.; Kulmala M.; Mohr C. Characterization of Offline Analysis of Particulate Matter with FIGAERO-CIMS. Atmos. Meas. Tech. 2023, 16 (5), 1147–1165. 10.5194/amt-16-1147-2023.

[ref21] Thornton J. A.; Mohr C.; Schobesberger S.; D’Ambro E. L.; Lee B. H.; Lopez-Hilfiker F. D. Evaluating Organic Aerosol Sources and Evolution with a Combined Molecular Composition and Volatility Framework Using the Filter Inlet for Gases and Aerosols (FIGAERO). Acc. Chem. Res. 2020, 53 (8), 1415–1426. 10.1021/acs.accounts.0c00259. 32648739

[ref22] Du M.; Voliotis A.; Shao Y.; Wang Y.; Bannan T. J.; Pereira K. L.; Hamilton J. F.; Percival C. J.; Alfarra M. R.; McFiggans G. Combined Application of Online FIGAERO-CIMS and Offline LC-Orbitrap Mass Spectrometry (MS) to Characterize the Chemical Composition of Secondary Organic Aerosol (SOA) in Smog Chamber Studies. Atmos. Meas. Tech. 2022, 15 (14), 4385–4406. 10.5194/amt-15-4385-2022.

[ref23] Romonosky D. E.; Li Y.; Shiraiwa M.; Laskin A.; Laskin J.; Nizkorodov S. A. Aqueous Photochemistry of Secondary Organic Aerosol of α-Pinene and α-Humulene Oxidized with Ozone, Hydroxyl Radical, and Nitrate Radical. J. Phys. Chem. A 2017, 121 (6), 1298–1309. 10.1021/acs.jpca.6b10900. 28099012

[ref24] Wong C.; Vite D.; Nizkorodov S. A. Stability of α-Pinene and d -Limonene Ozonolysis Secondary Organic Aerosol Compounds Toward Hydrolysis and Hydration. ACS Earth Space Chem. 2021, 5 (10), 2555–2564. 10.1021/acsearthspacechem.1c00171.

[ref25] Bateman A. P.; Walser M. L.; Desyaterik Y.; Laskin J.; Laskin A.; Nizkorodov S. A. The Effect of Solvent on the Analysis of Secondary Organic Aerosol Using Electrospray Ionization Mass Spectrometry. Environ. Sci. Technol. 2008, 42 (19), 7341–7346. 10.1021/es801226w. 18939568

[ref26] Chen K.; Raeofy N.; Lum M.; Mayorga R.; Woods M.; Bahreini R.; Zhang H.; Lin Y. H. Solvent Effects on Chemical Composition and Optical Properties of Extracted Secondary Brown Carbon Constituents. Aerosol Sci. Technol. 2022, 56 (10), 917–930. 10.1080/02786826.2022.2100734.

[ref27] Resch J.; Wolfer K.; Barth A.; Kalberer M. Effects of Storage Conditions on the Molecular-Level Composition of Organic Aerosol Particles. Atmos. Chem. Phys. 2023, 23 (16), 9161–9171. 10.5194/acp-23-9161-2023.

[ref28] Keller A.; Kalbermatter D. M.; Wolfer K.; Specht P.; Steigmeier P.; Resch J.; Kalberer M.; Hammer T.; Vasilatou K. The Organic Coating Unit, an All-in-One System for Reproducible Generation of Secondary Organic Matter Aerosol. Aerosol Sci. Technol. 2022, 56 (10), 947–958. 10.1080/02786826.2022.2110448.

[ref29] Smith C. A.; Want E. J.; O’Maille G.; Abagyan R.; Siuzdak G. XCMS: Processing Mass Spectrometry Data for Metabolite Profiling Using Nonlinear Peak Alignment, Matching, and Identification. Anal. Chem. 2006, 78 (3), 779–787. 10.1021/ac051437y. 16448051

[ref30] Tautenhahn R.; Bottcher C.; Neumann S. Highly Sensitive Feature Detection for High Resolution LC/MS. BMC Bioinf. 2008, 9, 504 10.1186/1471-2105-9-504. PMC2639432 19040729

[ref31] Benton H. P.; Want E. J.; Ebbels T. M. D. Correction of Mass Calibration Gaps in Liquid Chromatography-Mass Spectrometry Metabolomics Data. Bioinformatics 2010, 26 (19), 2488–2489. 10.1093/bioinformatics/btq441. 20671148

[ref32] Hakola H.; Hellén H.; Tarvainen V.; Bäck J.; Patokoski J.; Rinne J. Annual Variations of Atmospheric VOC Concentrations in a Boreal Forest. Boreal Environ. Res. 2009, 14 (4), 722–730.

[ref33] Bäck J.; Aalto J.; Henriksson M.; Hakola H.; He Q.; Boy M. Chemodiversity of a Scots Pine Stand and Implications for Terpene Air Concentrations. Biogeosciences 2012, 9 (2), 689–702. 10.5194/bg-9-689-2012.

[ref34] Guenther A. B.; Jiang X.; Heald C. L.; Sakulyanontvittaya T.; Duhl T.; Emmons L. K.; Wang X. The Model of Emissions of Gases and Aerosols from Nature Version 2.1 (MEGAN2.1): An Extended and Updated Framework for Modeling Biogenic Emissions. Geosci. Model Dev. 2012, 5 (6), 1471–1492. 10.5194/gmd-5-1471-2012.

[ref35] Kenseth C. M.; Huang Y.; Zhao R.; Dalleska N. F.; Caleb Hethcox J.; Stoltz B. M.; Seinfeld J. H. Synergistic O 3 + OH Oxidation Pathway to Extremely Low-Volatility Dimers Revealed in β-Pinene Secondary Organic Aerosol. Proc. Natl. Acad. Sci. U.S.A. 2018, 115 (33), 8301–8306. 10.1073/pnas.1804671115. 30076229 PMC6099901

[ref36] Sato K.; Jia T.; Tanabe K.; Morino Y.; Kajii Y.; Imamura T. Terpenylic Acid and Nine-Carbon Multifunctional Compounds Formed during the Aging of β-Pinene Ozonolysis Secondary Organic Aerosol. Atmos. Environ. 2016, 130, 127–135. 10.1016/j.atmosenv.2015.08.047.

[ref37] Zhao R.; Kenseth C. M.; Huang Y.; Dalleska N. F.; Kuang X. M.; Chen J.; Paulson S. E.; Seinfeld J. H. Rapid Aqueous-Phase Hydrolysis of Ester Hydroperoxides Arising from Criegee Intermediates and Organic Acids. J. Phys. Chem. A 2018, 122 (23), 5190–5201. 10.1021/acs.jpca.8b02195. 29782168

[ref38] Kourtchev I.; Giorio C.; Manninen A.; Wilson E.; Mahon B.; Aalto J.; Kajos M.; Venables D.; Ruuskanen T.; Levula J.; Loponen M.; Connors S.; Harris N.; Zhao D.; Kiendler-Scharr A.; Mentel T.; Rudich Y.; Hallquist M.; Doussin J. F.; Maenhaut W.; Bäck J.; Petäjä T.; Wenger J.; Kulmala M.; Kalberer M. Enhanced Volatile Organic Compounds Emissions and Organic Aerosol Mass Increase the Oligomer Content of Atmospheric Aerosols. Sci. Rep. 2016, 6, 35038 10.1038/srep35038. 27733773 PMC5062071

[ref39] Zhang X.; McVay R. C.; Huang D. D.; Dalleska N. F.; Aumont B.; Flagan R. C.; Seinfeld J. H. Formation and Evolution of Molecular Products in $α$-Pinene Secondary Organic Aerosol. Proc. Natl. Acad. Sci. U.S.A. 2015, 112 (46), 14168–14173. 10.1073/pnas.1517742112. 26578760 PMC4655512

[ref40] Maben H. K.; Ziemann P. J. Kinetics of Oligomer-Forming Reactions Involving the Major Functional Groups Present in Atmospheric Secondary Organic Aerosol Particles. Environ. Sci. Process. Impacts 2023, 25, 214–228. 10.1039/D2EM00124A. 35665793

[ref41] Claflin M. S.; Krechmer J. E.; Hu W.; Jimenez J. L.; Ziemann P. J. Functional Group Composition of Secondary Organic Aerosol Formed from Ozonolysis of α-Pinene under High VOC and Autoxidation Conditions. ACS Earth Space Chem. 2018, 2 (11), 1196–1210. 10.1021/acsearthspacechem.8b00117.

[ref42] Pospisilova V.; Lopez-Hilfiker F. D.; Bell D. M.; El Haddad I.; Mohr C.; Huang W.; Heikkinen L.; Xiao M.; Dommen J.; Prevot A. S. H.; Baltensperger U.; Slowik J. G. On the Fate of Oxygenated Organic Molecules in Atmospheric Aerosol Particles. Sci. Adv. 2020, 6 (11), eaax8922 10.1126/sciadv.aax8922. 32201715 PMC7069715

[ref43] Vaden T. D.; Imre D.; Beránek J.; Shrivastava M.; Zelenyuk A. Evaporation Kinetics and Phase of Laboratory and Ambient Secondary Organic Aerosol. Proc. Natl. Acad. Sci. U.S.A. 2011, 108 (6), 2190–2195. 10.1073/pnas.1013391108. 21262848 PMC3038757

[ref44] Wilson J.; Imre D.; Beránek J.; Shrivastava M.; Zelenyuk A. Evaporation Kinetics of Laboratory-Generated Secondary Organic Aerosols at Elevated Relative Humidity. Environ. Sci. Technol. 2015, 49 (1), 243–249. 10.1021/es505331d. 25494490

[ref45] Li Z.; Tikkanen O. P.; Buchholz A.; Hao L.; Kari E.; Yli-Juuti T.; Virtanen A. Effect of Decreased Temperature on the Evaporation of α-Pinene Secondary Organic Aerosol Particles. ACS Earth Space Chem. 2019, 3 (12), 2775–2785. 10.1021/acsearthspacechem.9b00240.

[ref46] Li Z.; Buchholz A.; Barreira L. M. F.; Ylisirniö A.; Hao L.; Pullinen I.; Schobesberger S.; Virtanen A. Isothermal Evaporation of α-Pinene Secondary Organic Aerosol Particles Formed under Low NOx and High NOx Conditions. Atmos. Chem. Phys. 2023, 23 (1), 203–220. 10.5194/acp-23-203-2023.

[ref47] Ylisirniö A.; Buchholz A.; Mohr C.; Li Z.; Barreira L.; Lambe A.; Faiola C.; Kari E.; Yli-Juuti T.; Nizkorodov S. A.; Worsnop D. R.; Virtanen A.; Schobesberger S. Composition and Volatility of Secondary Organic Aerosol (SOA) Formed from Oxidation of Real Tree Emissions Compared to Simplified Volatile Organic Compound (VOC) Systems. Atmos. Chem. Phys. 2020, 20 (9), 5629–5644. 10.5194/acp-20-5629-2020.

[ref48] Yasmeen F.; Vermeylen R.; Szmigielski R.; Iinuma Y.; Böge O.; Herrmann H.; Maenhaut W.; Claeys M. Terpenylic Acid and Related Compounds: Precursors for Dimers in Secondary Organic Aerosol from the Ozonolysis of α- and β-Pinene. Atmos. Chem. Phys. 2010, 10 (19), 9383–9392. 10.5194/acp-10-9383-2010.

[ref49] Kourtchev I.; Doussin J. F.; Giorio C.; Mahon B.; Wilson E. M.; Maurin N.; Pangui E.; Venables D. S.; Wenger J. C.; Kalberer M. Molecular Composition of Fresh and Aged Secondary Organic Aerosol from a Mixture of Biogenic Volatile Compounds: A High-Resolution Mass Spectrometry Study. Atmos. Chem. Phys. 2015, 15 (10), 5683–5695. 10.5194/acp-15-5683-2015.

[ref50] Glasius M.; Lahaniati M.; Calogirou A.; Di Bella D.; Jensen N. R.; Hjorth J.; Kotzias D.; Larsen B. R. Carboxylic Acids in Secondary Aerosols from Oxidation of Cyclic Monoterpenes by Ozone. Environ. Sci. Technol. 2000, 34 (6), 1001–1010. 10.1021/es990445r.

[ref51] Witkowski B.; al-Sharafi M.; Błaziak K.; Gierczak T. Aging of α-Pinene Secondary Organic Aerosol by Hydroxyl Radicals in the Aqueous Phase: Kinetics and Products. Environ. Sci. Technol. 2023, 57 (15), 6040–6051. 10.1021/acs.est.2c07630. 37014140 PMC10116591

[ref52] Yasmeen F.; Vermeylen R.; Maurin N.; Perraudin E.; Doussin J. F.; Claeys M. Characterisation of Tracers for Aging of α-Pinene Secondary Organic Aerosol Using Liquid Chromatography/Negative Ion Electrospray Ionisation Mass Spectrometry. Environ. Chem. 2012, 9 (3), 236–246. 10.1071/EN11148.

[ref53] Yasmeen F.; Szmigielski R.; Vermeylen R.; Gõmez-González Y.; Surratt J. D.; Chan A. W. H.; Seinfeld J. H.; Maenhaut W.; Claeys M. Mass Spectrometric Characterization of Isomeric Terpenoic Acids from the Oxidation of α-Pinene, β-Pinene, d-Limonene, and Î″ 3-Carene in Fine Forest Aerosol. J. Mass Spectrom. 2011, 46 (4), 425–442. 10.1002/jms.1911. 21438093

[ref54] Claeys M.; Iinuma Y.; Szmigielski R.; Surratt J. D.; Blockhuys F.; Van Alsenoy C.; Böge O.; Sierau B.; Gómez-González Y.; Vermeylen R.; Van Der Veken P.; Shahgholi M.; Chan A. W. H.; Herrmann H.; Seinfeld J. H.; Maenhaut W. Terpenylic Acid and Related Compounds from the Oxidation of α-Pinene: Implications for New Particle Formation and Growth above Forests. Environ. Sci. Technol. 2009, 43 (18), 6976–6982. 10.1021/es9007596. 19806730

[ref55] Kahnt A.; Vermeylen R.; Iinuma Y.; Safi Shalamzari M.; Maenhaut W.; Claeys M. High-Molecular-Weight Esters in α-Pinene Ozonolysis Secondary Organic Aerosol: Structural Characterization and Mechanistic Proposal for Their Formation from Highly Oxygenated Molecules. Atmos. Chem. Phys. 2018, 18 (11), 8453–8467. 10.5194/acp-18-8453-2018.

[ref56] Beck M.; Hoffmann T. A Detailed MSn Study for the Molecular Identification of a Dimer Formed from Oxidation of Pinene. Atmos. Environ. 2016, 130, 120–126. 10.1016/j.atmosenv.2015.09.012.

[ref57] Kristensen K.; Enggrob K. L.; King S. M.; Worton D. R.; Platt S. M.; Mortensen R.; Rosenoern T.; Surratt J. D.; Bilde M.; Goldstein A. H.; Glasius M. Formation and Occurrence of Dimer Esters of Pinene Oxidation Products in Atmospheric Aerosols. Atmos. Chem. Phys. 2013, 13 (7), 3763–3776. 10.5194/acp-13-3763-2013.

[ref58] Claflin M. S.; Liu J.; Russell L. M.; Ziemann P. J. Comparison of Methods of Functional Group Analysis Using Results from Laboratory and Field Aerosol Measurements. Aerosol Sci. Technol. 2021, 55 (9), 1042–1058. 10.1080/02786826.2021.1918325.

[ref59] Hall W. A.; Johnston M. V. Oligomer Formation Pathways in Secondary Organic Aerosol from MS and MS/MS Measurements with High Mass Accuracy and Resolving Power. J. Am. Soc. Mass Spectrom. 2012, 23 (6), 1097–1108. 10.1007/s13361-012-0362-6. 22476934

[ref60] Kenseth C. M.; Hafeman N. J.; Rezgui S. P.; Chen J.; Huang Y.; Dalleska N. F.; Kjaergaard H. G.; Stoltz B. M.; Seinfeld J. H.; Wennberg P. O. Particle-Phase Accretion Forms Dimer Esters in Pinene Secondary Organic Aerosol. Science 2023, 382 (6672), 787–792. 10.1126/science.adi0857. 37972156

