# Supplementary material for: Prolonged Dark Chemical Processes in Secondary Organic Aerosols on Filters and in Aqueous Solution
Source: Environ Sci Technol. 2024 Jul 30;58(32):14318–28. doi: 10.1021/acs.est.4c01647 (PMC11325657; doi:10.1021/acs.est.4c01647)
Supplement: Supplementary file 1 — es4c01647_si_001.pdf [file es4c01647_si_001.pdf]

Supporting Information

Prolonged dark chemical processes in secondary organic aerosols on filters and in aqueous solution

Julian Resch[a], Kangwei Li[a] and Markus Kalberer[a]*

Department of Environmental Sciences, University of Basel, Basel 4056, Switzerland

Correspondence to: Markus Kalberer (Markus.Kalberer@unibas.ch)

This PDF file includes 25 pages:

Fig. S1 to S29

Table S1 and S2

References (1-9)

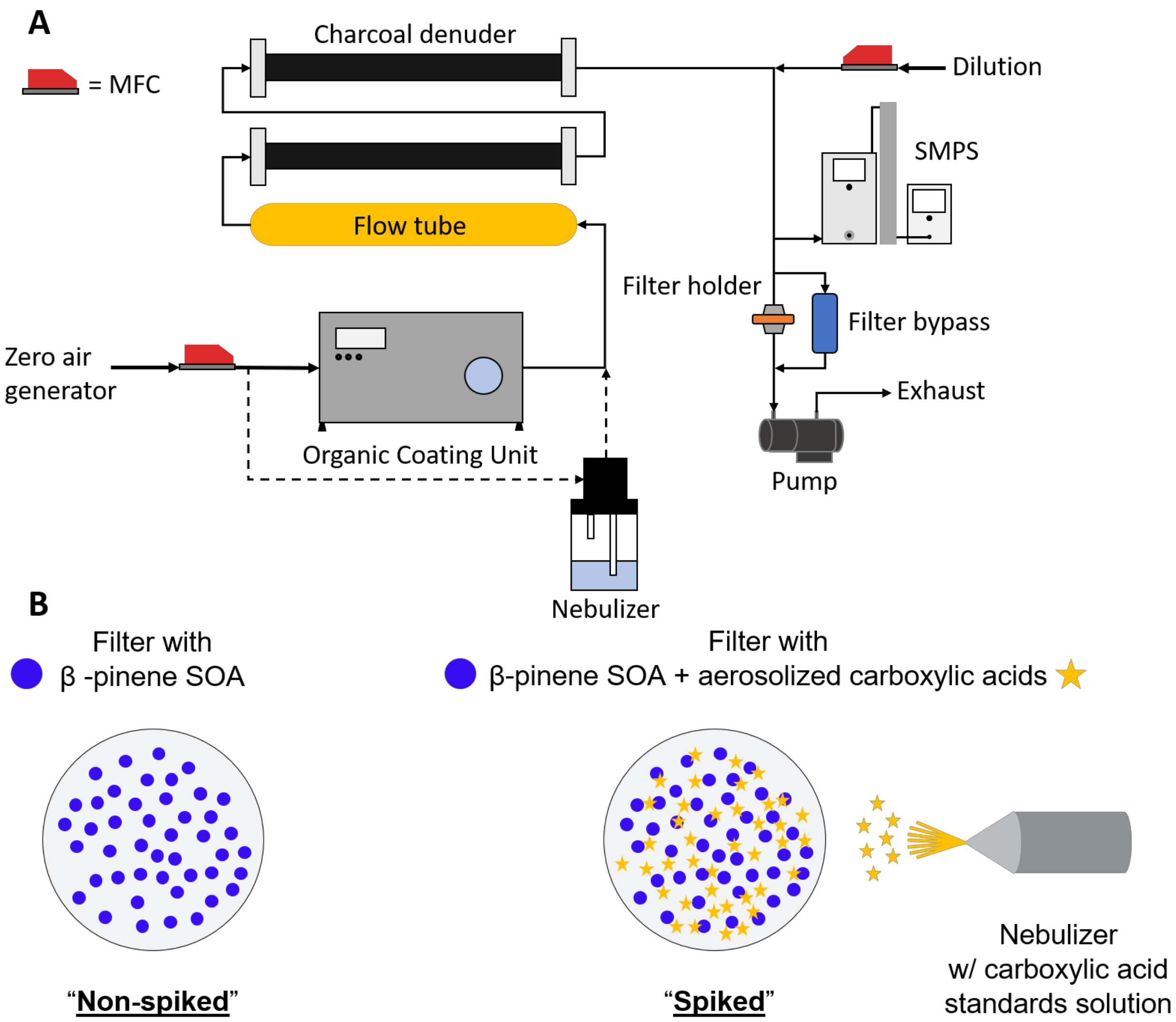

Figure S 1: (A) Setup used for the collection of laboratory-generated β-pinene secondary organic aerosol samples as described in Resch et al., (2023)[1].(B) For "spiking" experiments a nebulizer was additionally used instead of the OCU to deposit carboxylic acid standards onto the previously collected β-pinene filters. Nebulizing a mixture of carboxylic acids standards onto the SOA filter assures that the carboxylic acids are deposited on the filters without a solvent, which could interfere with the SOA particle phase reaction studied.

Table S 1: Complete list containing Compound ID, observed m/z in negative polarity mode, Molecular Formula and literature references for all dimer esters investigated.

Compound ID[reference]	Observed m/z [-]	Molecular Formula
M_W302[2]	301.16512	$C_{15}H_{26}O_6$
M_W 312[2]	311.1504	$C_{16}H_{24}O_6$
M_W 314[2]	313.1651	$C_{16}H_{26}O_6$
M_W 316[2]	315.1443	$C_{15}H_{24}O_7$
M_W 324	323.1860	$C_{18}H_{28}O_5$
M_W 328[2]	327.1444	$C_{16}H_{24}O_7$
M_W 330[2]	329.1600	$C_{16}H_{26}O_7$
M_W 332[2]	331.1393	$C_{15}H_{24}O_8$
M_W 336[2]	335.1859	$C_{19}H_{28}O_5$
M_W 338[2,3,4]	337.2015	$C_{19}H_{30}O_5$
M_W 340[2]	339.1808	$C_{18}H_{28}O_6$
M_W 342[2]	341.1600	$C_{17}H_{26}O_7$
M_W 344a[2,4,5,6,7]	343.1393	$C_{16}H_{24}O_8$
M_W 344b[2]	343.1756	$C_{17}H_{28}O_7$
M_W 352[3]	351.1828	$C_{19}H_{28}O_6$
M_W 354[2]	353.1964	$C_{19}H_{30}O_6$
M_W 356[2,3]	355.1766	$C_{18}H_{28}O_7$
M_W 358[2,4,5,6,7]	357.1558	$C_{17}H_{26}O_8$
M_W 360[2]	359.1715	$C_{17}H_{28}O_8$
M_W 362[2]	361.1508	$C_{16}H_{26}O_9$
M_W 368[2,4,5,6,8]	367.1764	$C_{19}H_{28}O_7$
M_W 370[2,3,4]	369.1921	$C_{19}H_{30}O_7$
M_W 372[2]	371.1714	$C_{18}H_{28}O_8$
M_W 374a[2]	373.1499	$C_{17}H_{26}O_9$
M_W 374b[3]	373.1851	$C_{18}H_{30}O_8$
M_W 378[2]	377.1432	$C_{16}H_{26}O_{10}$
M_W 384[2]	383.1715	$C_{19}H_{28}O_8$
M_W 386[2]	385.1872	$C_{19}H_{30}O_8$
M_W 388a[2]	387.1665	$C_{18}H_{28}O_9$
M_W 388b[4,5]	387.2022	$C_{19}H_{32}O_8$
M_W 400[2]	399.1663	$C_{19}H_{28}O_9$
M_W 406[2,3]	405.1770	$C_{18}H_{30}O_{10}$
M_W 420[3]	419.1525	$C_{18}H_{28}O_{11}$

References: (2) Kristensen et al., (2016); (3) Kenseth et al., (2018); (4) Kourtchev et al., (2015); (5) Kristensen et al., (2014); (6) Yasmeen et al., (2010); (7) Sato et al., (2016); (8) Müller et al., (2008).

Table S 2: List of oligomers analyzed. Molecular weight, tentative chemical formula, retention time and sample type of highest observed peak are given.

Compound ID	Observed m/z (-)	Molecular formula	Retention time [min]	Sample type of highest observed peak
MW338	337.2015	C19H30O5	20.03	Both
MW340	339.1808	C18H28O6	16.77	Aged
			18.54	Fresh
MW344a	343.1393	C16H24O8	13.51	Aged
MW344b	343.1757	C17H28O7	16.74	Aged
			16.34	Fresh
			16.58	Fresh
MW354	353.1964	C19H30O6	21.17	Aged
			22.23	Fresh
MW356	355.1757	C18H28O7	17.48	Aged
			15.72	Fresh
MW360	359.1706	C17H28O8	17.57	Aged
MW362	361.1499	C16H26O9	14.68	Both
MW368	367.1757	C19H28O7	13.83	Aged
MW370	369.1913	C19H30O7	17.66	Aged
			18.54	Fresh
MW372	371.1706	C18H28O8	14.87	Aged
			15.64	Fresh
MW386	385.1862	C19H30O8	15.05	Both
MW465	464.2585	C29H36O5	18.42	/
MW471	470.2095	C30H30O5	18.47	/
MW479	478.2355	C29H34O6	15.67	/
MW494	493.2294	C22H37O12	18.53	/
MW514	513.1954	C24H33O12	17.72	/
MW572	571.3123	C29H47O11	17.90	/

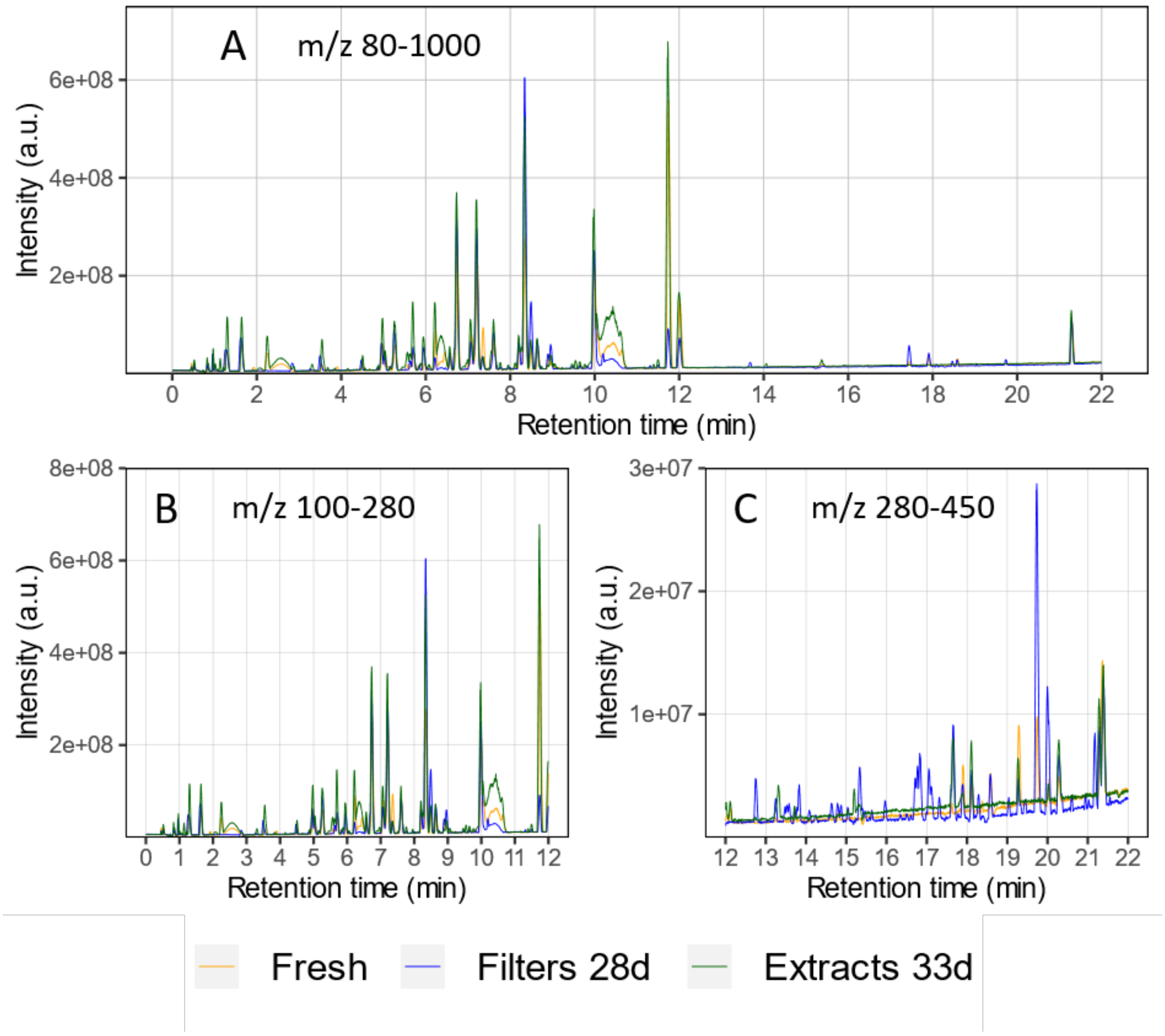

Figure S 2: (A) BPC representing fresh and aged filter and extract samples with m/z 80-1000. (B) BPC of the monomer region with m/z 100-280 between 0 and 12 minutes. The signal intensity is increased for almost all peaks in the 33-day old extracts compared to the fresh samples and stored filters. (C) BPC of the dimer region with m/z 280-450 between 12 and 22 minutes. The 28-day old filter samples show a significant increase in both intensity and number of peaks compared to the fresh samples and aged extracts. A higher background (assigned to an unknown compound detected at m/z 305.0230, which is a constant background signal in our system) between peaks is observed for the stored extract samples. This increase in the base peak background leads to an increase of TIC signal in the stored extracts in Figure 2 (C), hence the signal intensity minus the background signal would be lower than the fresh samples.

Highest intensity dimers in 28-day old filters

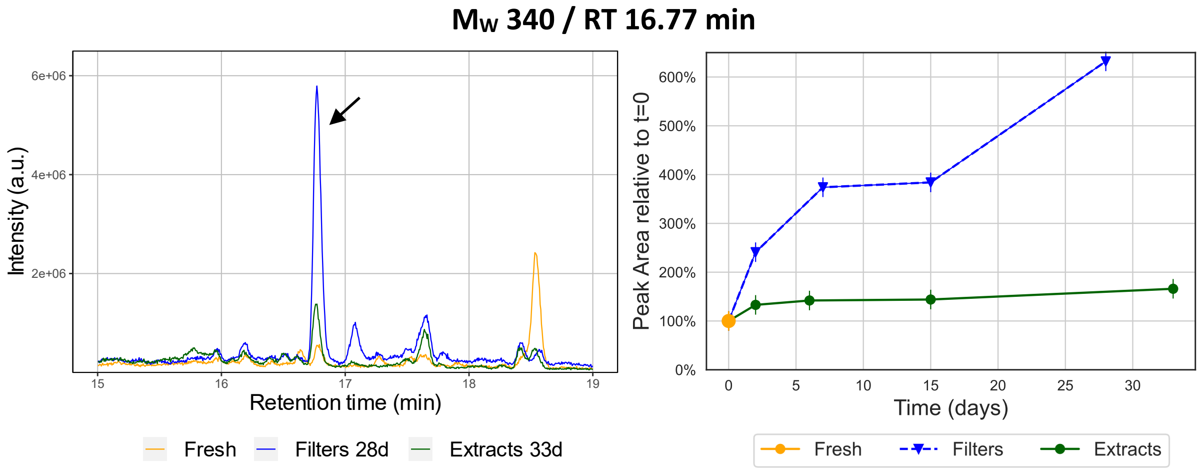

Figure S 3: EIC of the m/z 339.1808 dimer ester with highest intensity in the 28-day old filter samples. The corresponding timeseries for this isomer is given on the right.

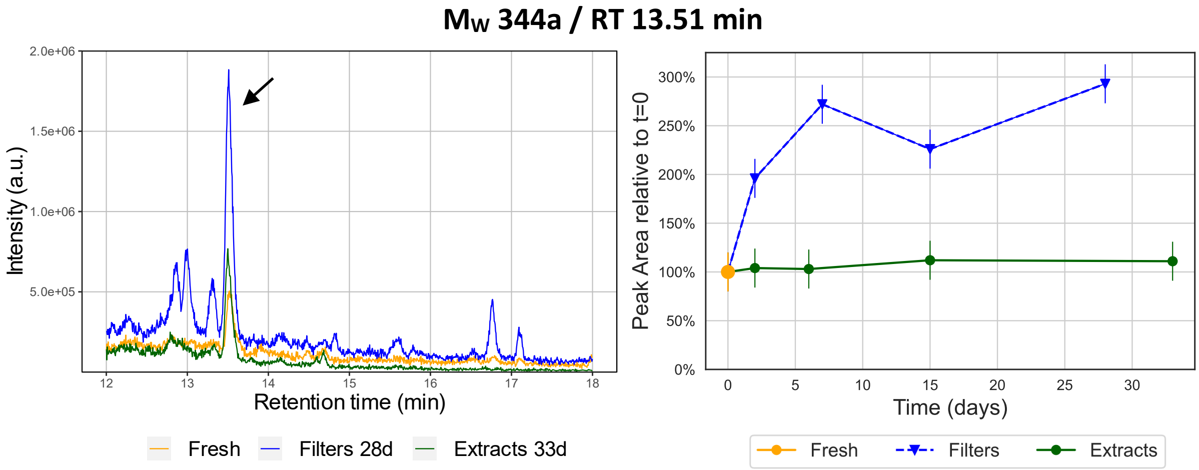

Figure S 4: EIC of the m/z 343.1393 dimer ester with highest intensity in the 28-day old filter samples. The corresponding timeseries for this isomer is given on the right.

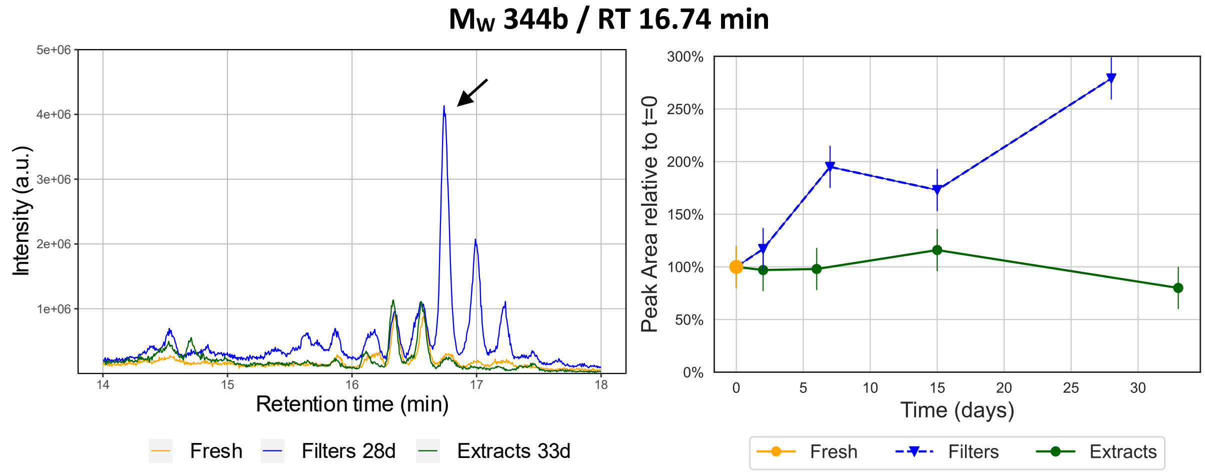

Figure S 5: EIC of the m/z 343.1757 dimer ester with highest intensity in the 28-day old filter samples. The corresponding timeseries for this isomer is given on the right.

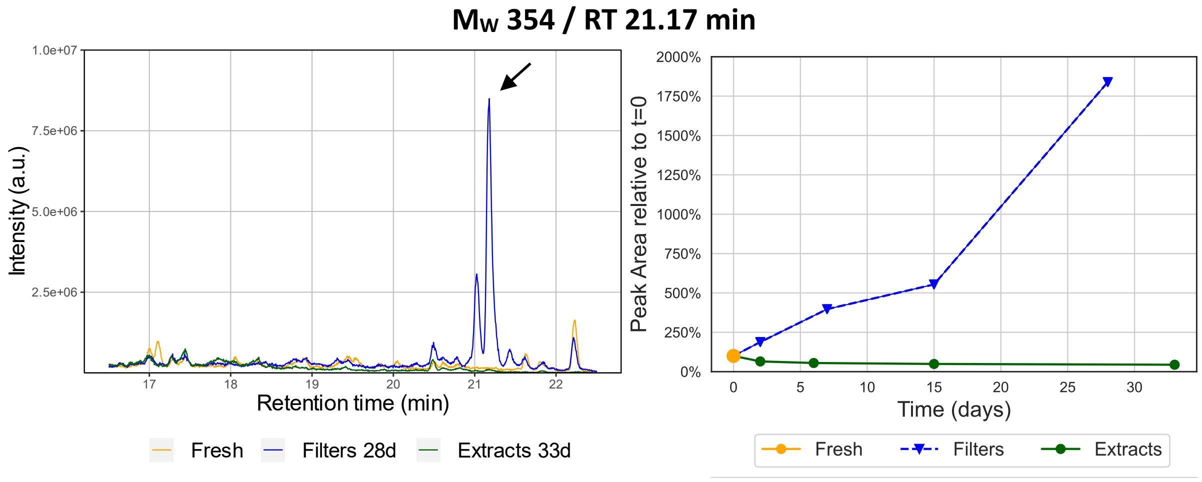

Figure S 6: EIC of the m/z 353.1964 dimer ester with highest intensity in the 28-day old filter samples. The corresponding timeseries for this isomer is given on the right.

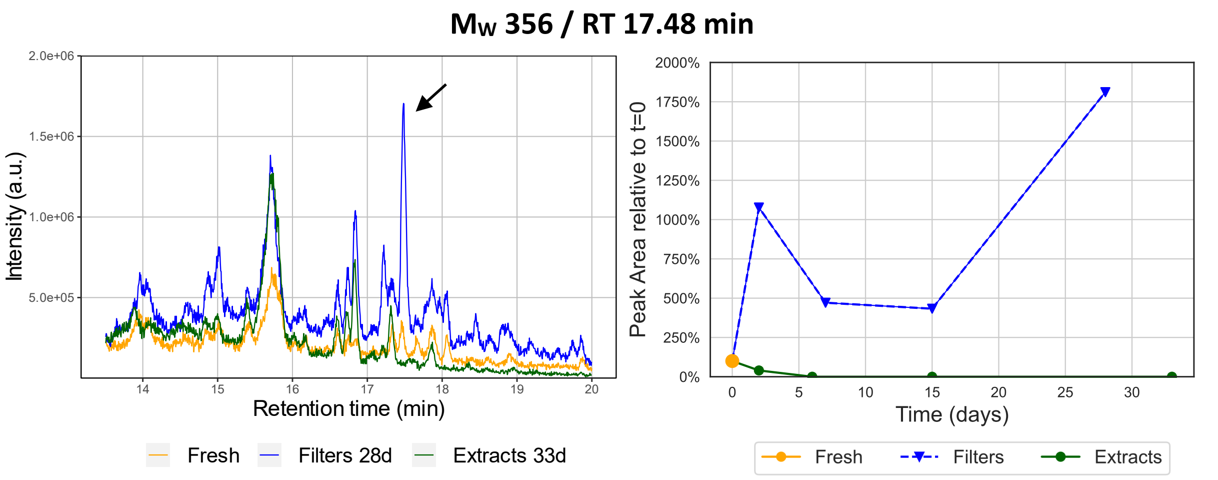

Figure S 7: EIC of the m/z 355.1757 dimer ester with highest intensity in the 28-day old filter samples. The corresponding timeseries for this isomer is given on the right.

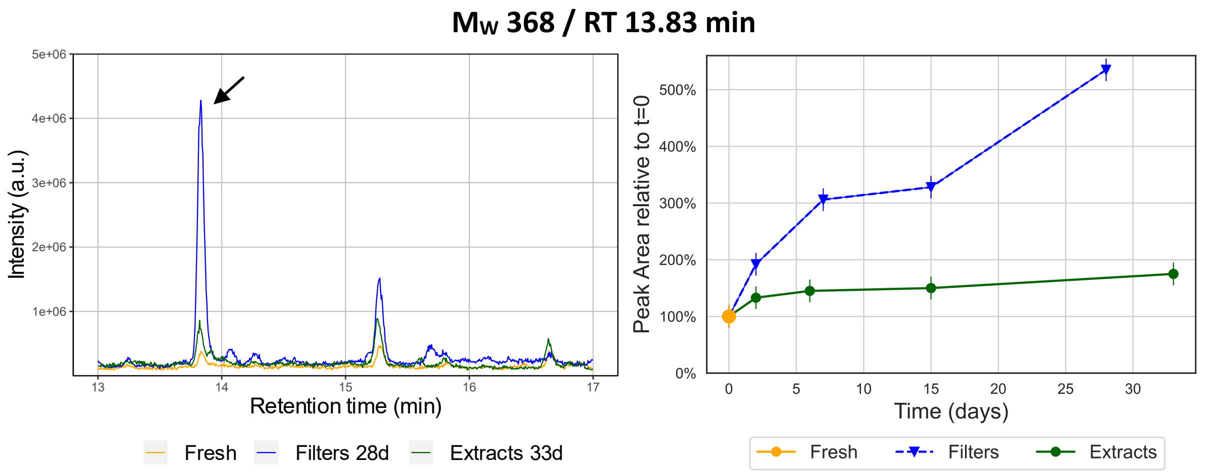

Figure S 8: EIC of the m/z 367.1757 dimer ester with highest intensity in the 28-day old filter samples. The corresponding timeseries for this isomer is given on the right.

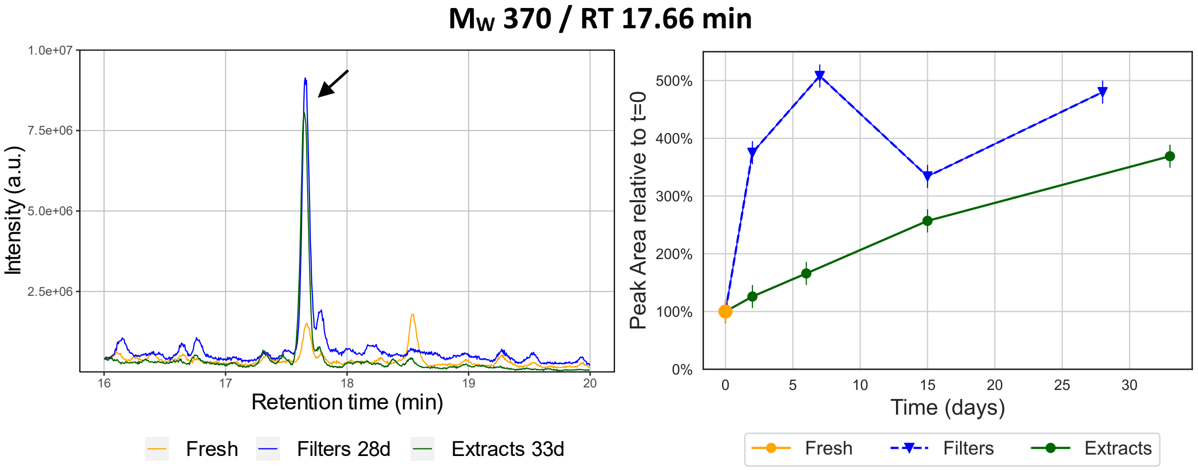

Figure S 9: EIC of the m/z 369.1913 dimer ester with highest intensity in the 28-day old filter samples. The corresponding timeseries for this isomer is given on the right.

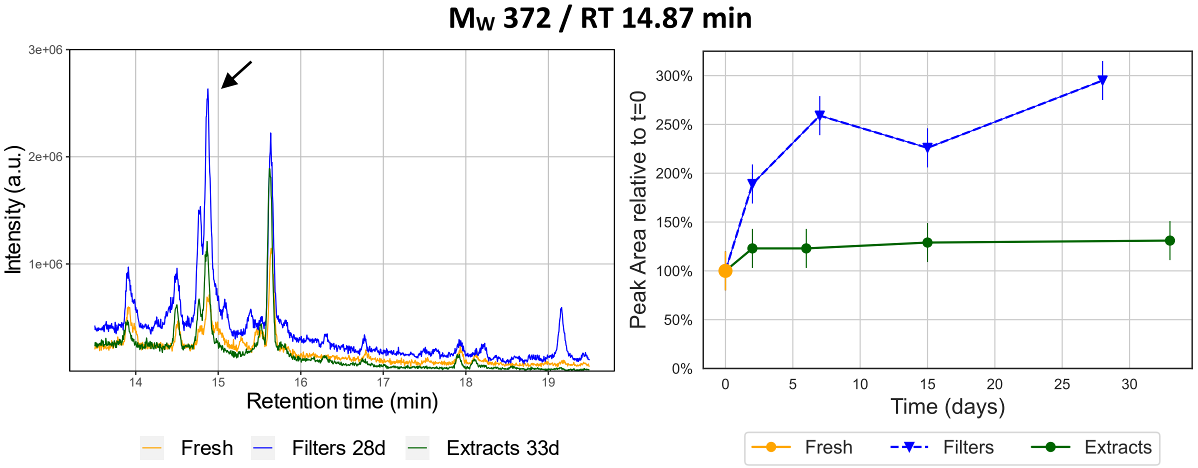

Figure S 10: EIC of the m/z 371.1706 dimer ester with highest intensity in the 28-day old filter samples. The corresponding timeseries for this isomer is given on the right.

Highest intensity dimers in fresh samples

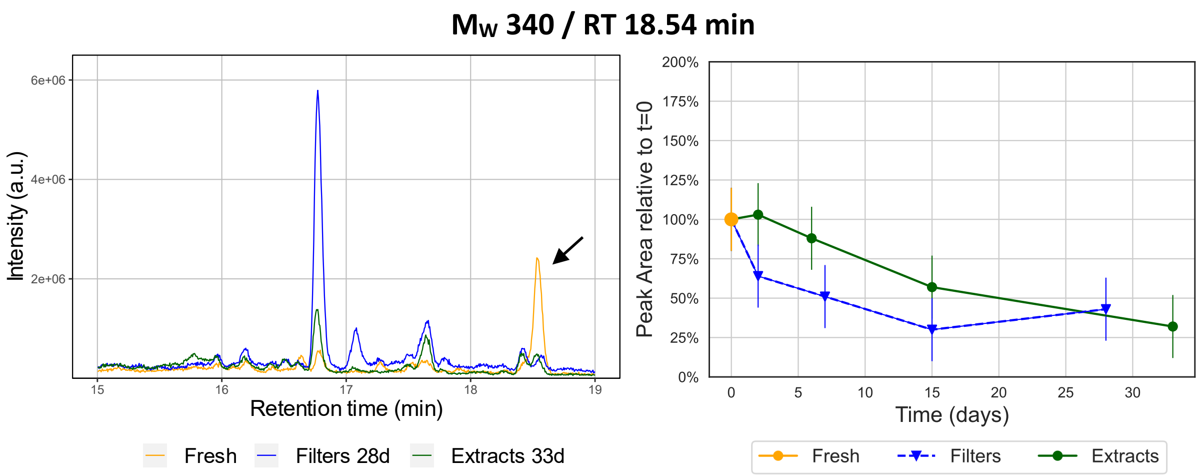

Figure S 11: EIC of the m/z 339.1808 dimer ester with highest intensity in the fresh samples. The corresponding timeseries for this isomer is given on the right.

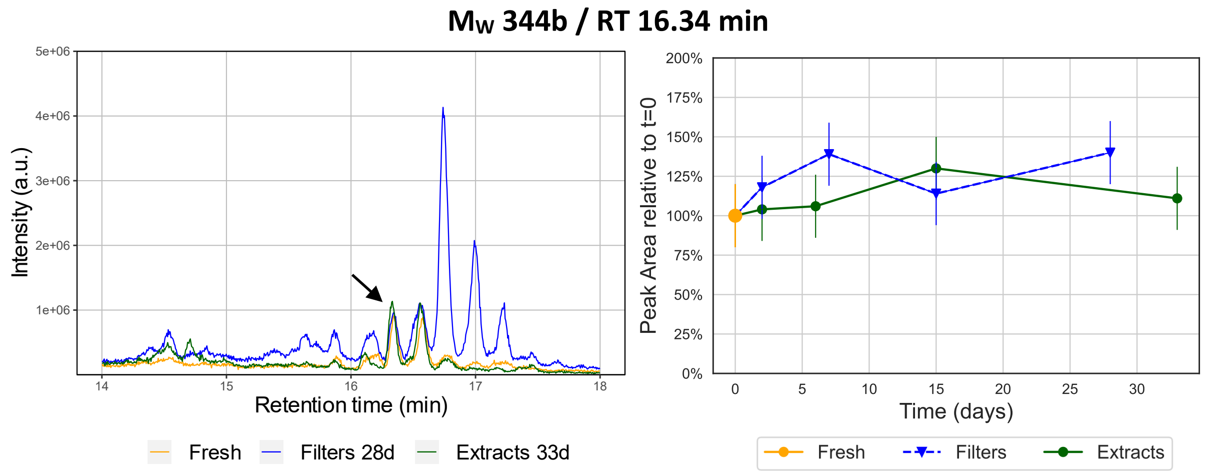

Figure S 12: EIC of the m/z 343.1757 dimer ester with highest intensity in the fresh samples. The corresponding timeseries for this isomer is given on the right.

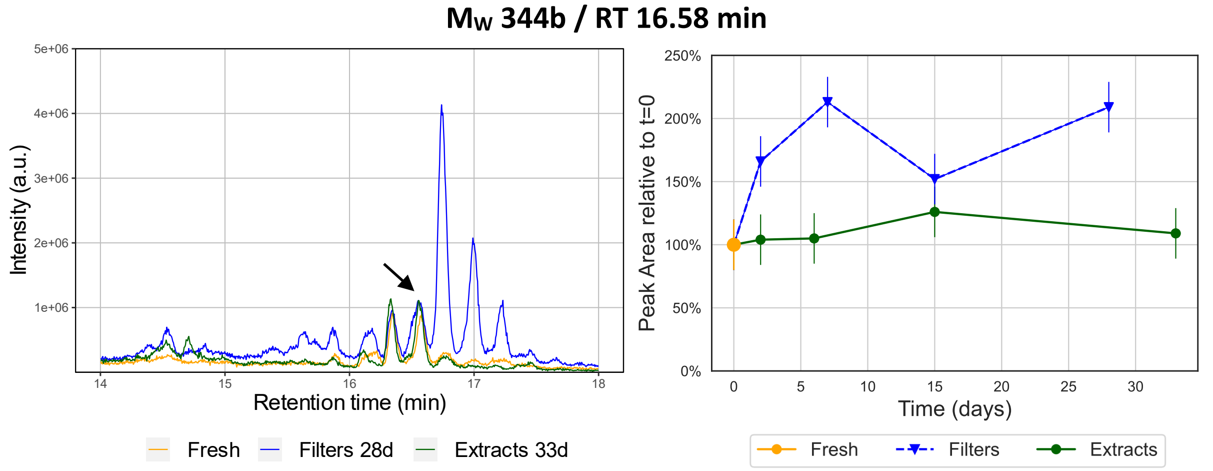

Figure S 13: EIC of the m/z 343.1757 dimer ester with highest intensity in the fresh samples. The corresponding timeseries for this isomer is given on the right.

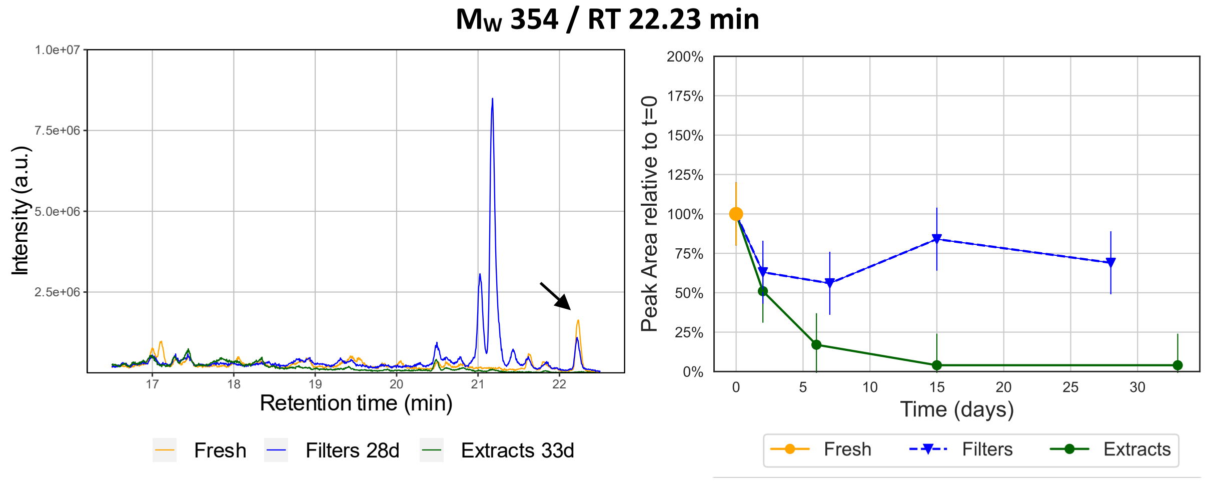

Figure S 14: EIC of the m/z 353.1964 dimer ester with highest intensity in the fresh samples. The corresponding timeseries for this isomer is given on the right.

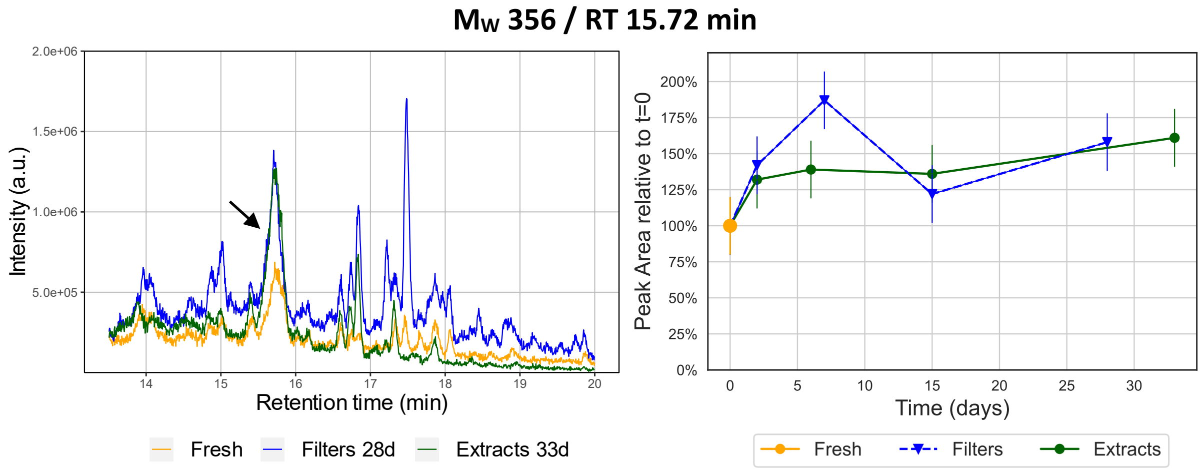

Figure S 15: EIC of the m/z 355.1757 dimer ester with highest intensity in the fresh samples. The corresponding timeseries for this isomer is given on the right.

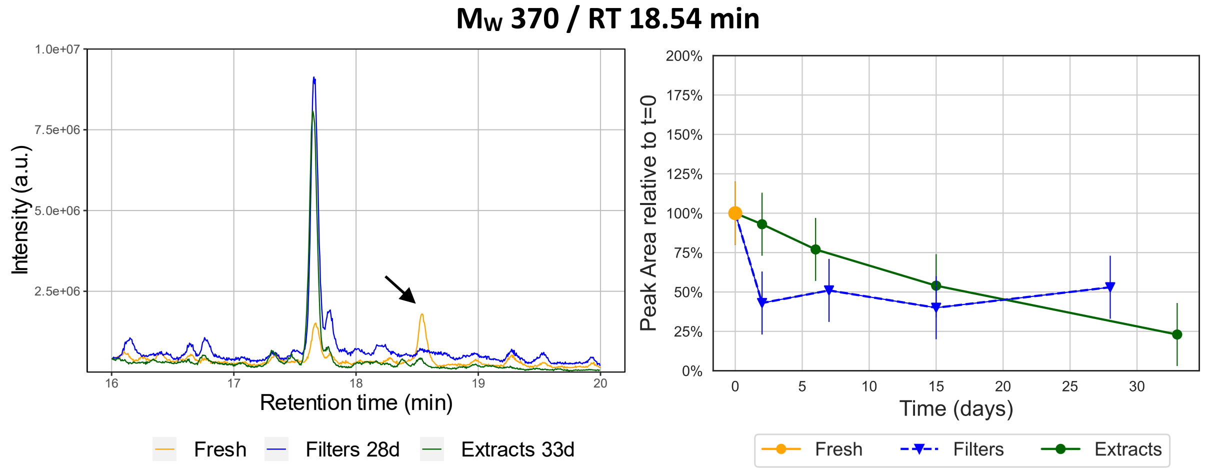

Figure S 16: EIC of the m/z 369.1913 dimer ester with highest intensity in the fresh samples. The corresponding timeseries for this isomer is given on the right.

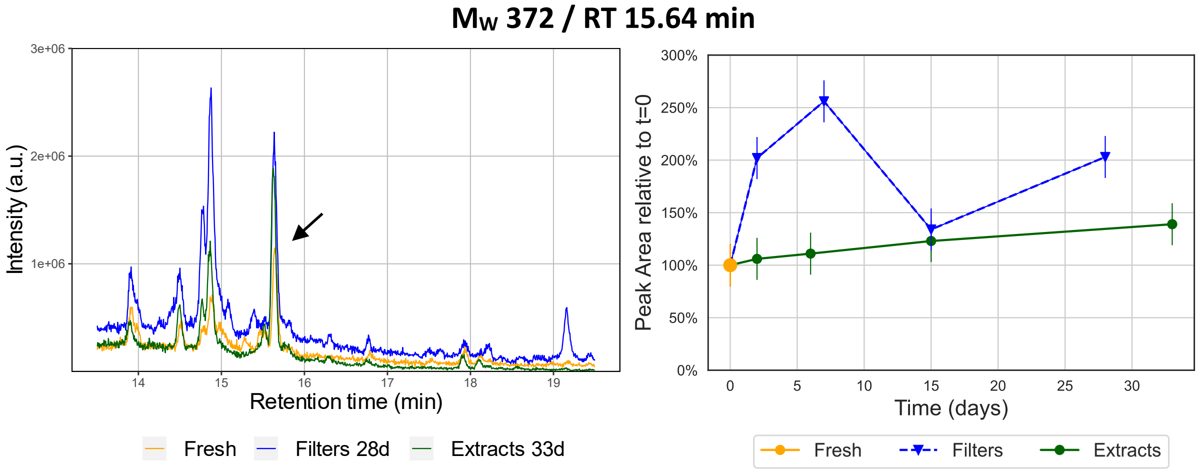

Figure S 17: EIC of the m/z 371.1706 dimer ester with highest intensity in the fresh samples.

The corresponding timeseries for this isomer is given on the right.

Highest intensity dimers in both fresh samples and 28-day old filter samples

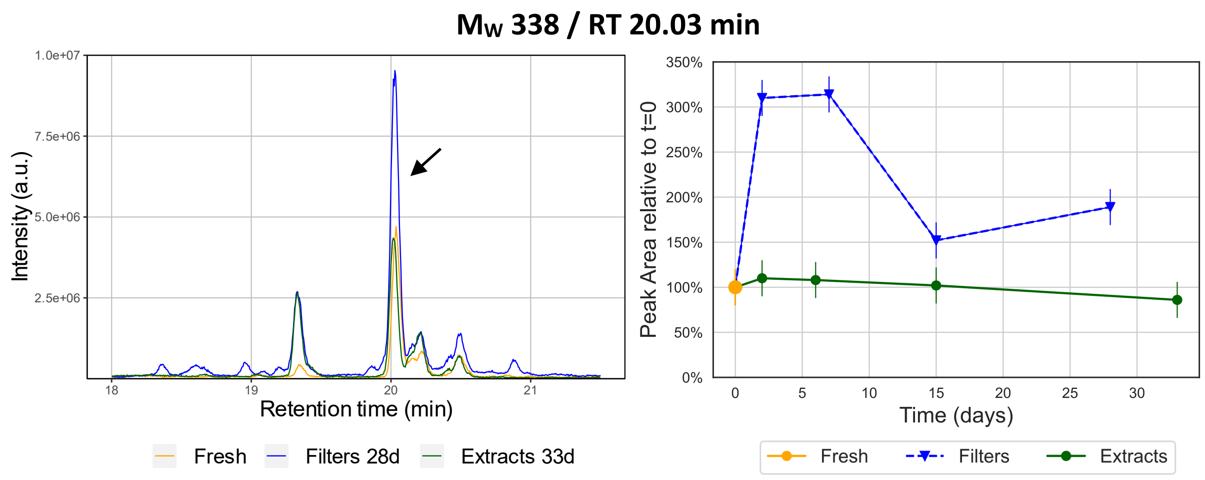

Figure S 18: EIC of the m/z 337.2015 dimer ester with highest intensity in both the fresh and 28-day old filter samples. The corresponding timeseries for this isomer is given on the right.

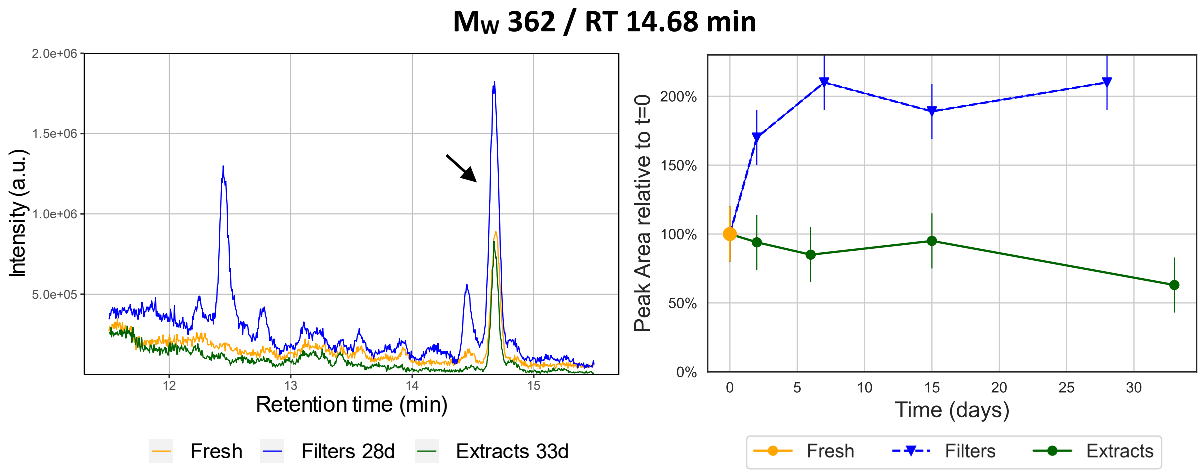

Figure S 19: EIC of the m/z 361.1499 dimer ester with highest intensity in both the fresh and 28-day old filter samples. The corresponding timeseries for this isomer is given on the right.

M_W 386 / RT 15.05 min

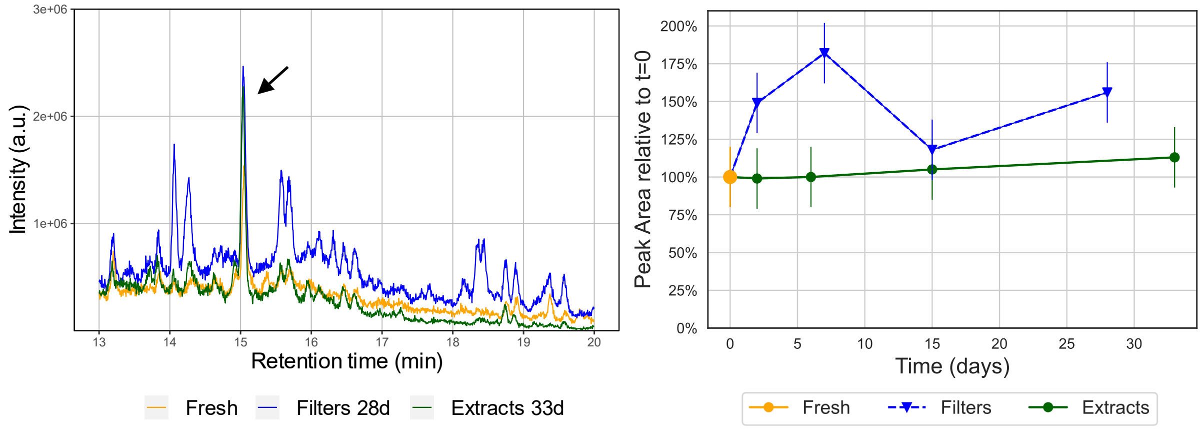

Figure S 20: EIC of the m/z 385.1862 dimer ester with highest intensity in both the fresh and 28-day old filter samples. The corresponding timeseries for this isomer is given on the right.

Trimer compounds investigated in this study:

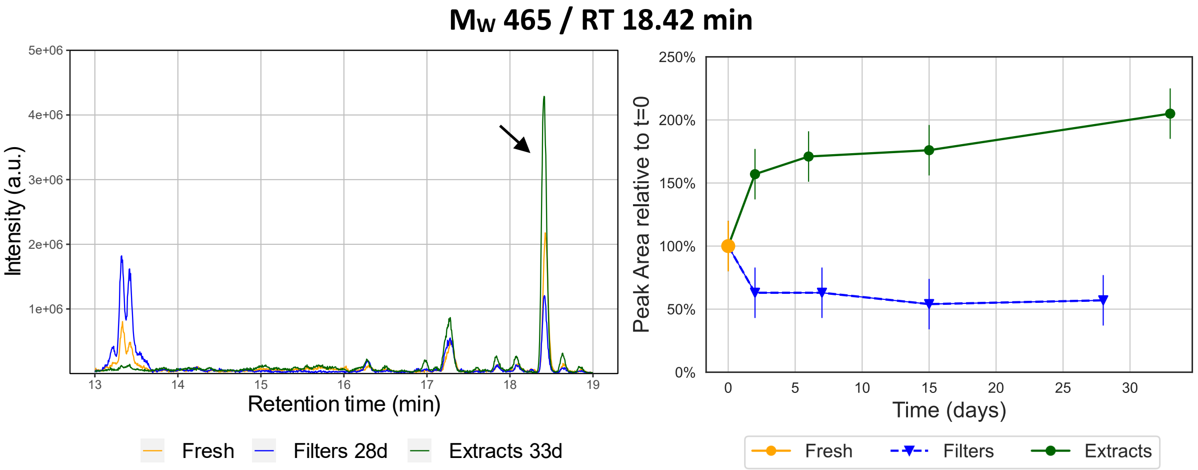

Figure S 21: EIC of the m/z 464.2585 compound tentatively assigned as a trimer. The corresponding timeseries of the isomer is given on the right.

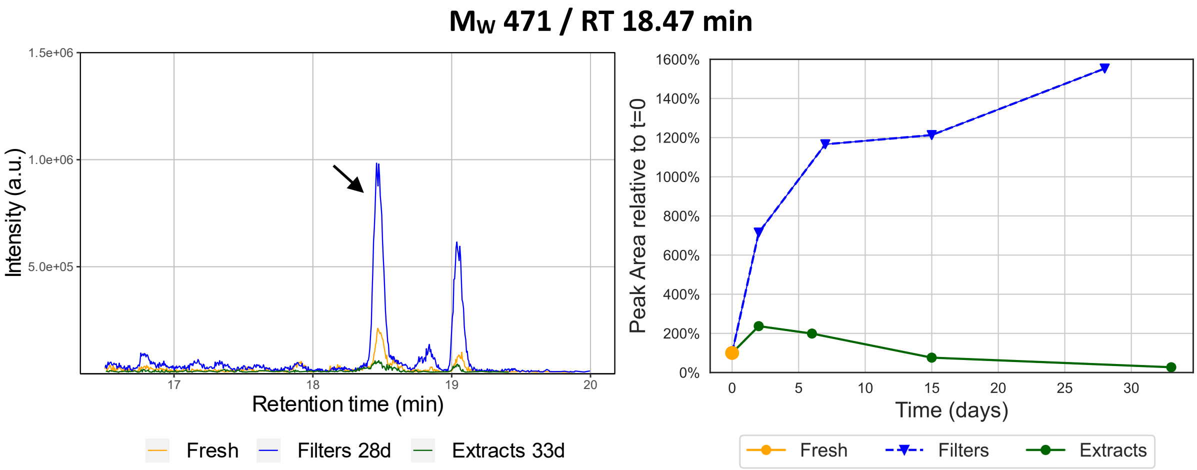

Figure S 22: EIC of the m/z 470.2095 compound tentatively assigned as a trimer. The corresponding timeseries of the isomer is given on the right.

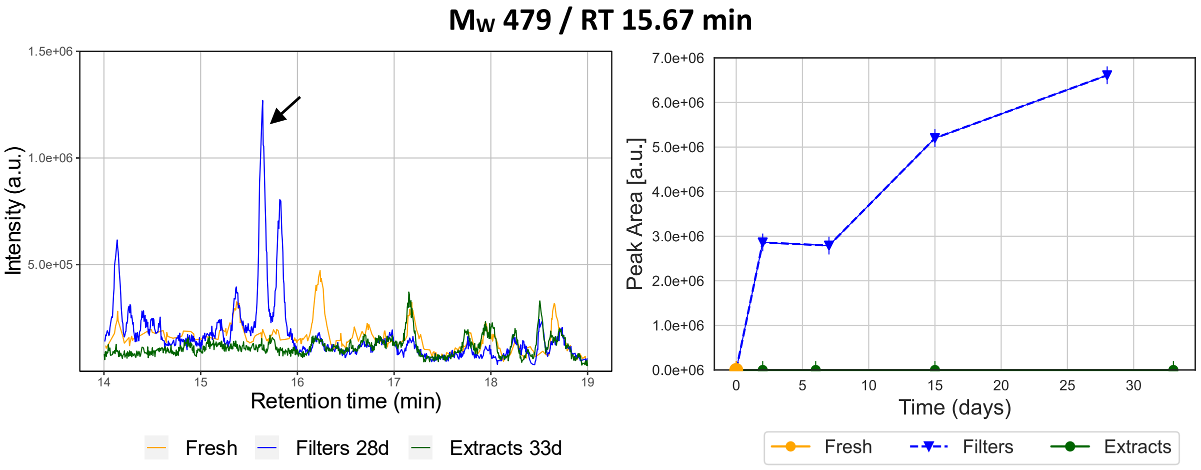

Figure S 23: EIC of the m/z 478.2331 compound tentatively assigned as a trimer. The corresponding timeseries of the isomer is given on the right.

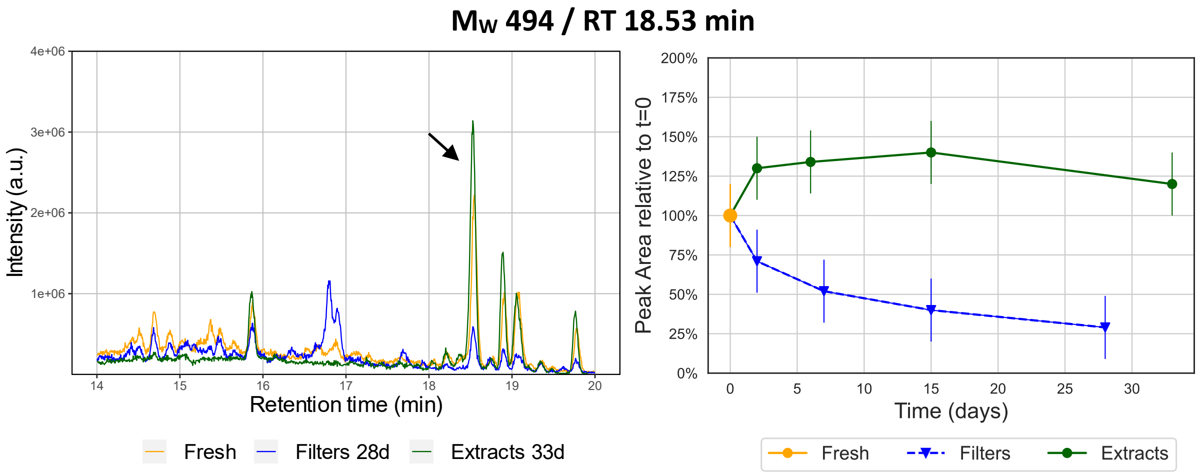

Figure S 24: EIC of the m/z 493.2294 compound tentatively assigned as a trimer. The corresponding timeseries of the isomer is given on the right.

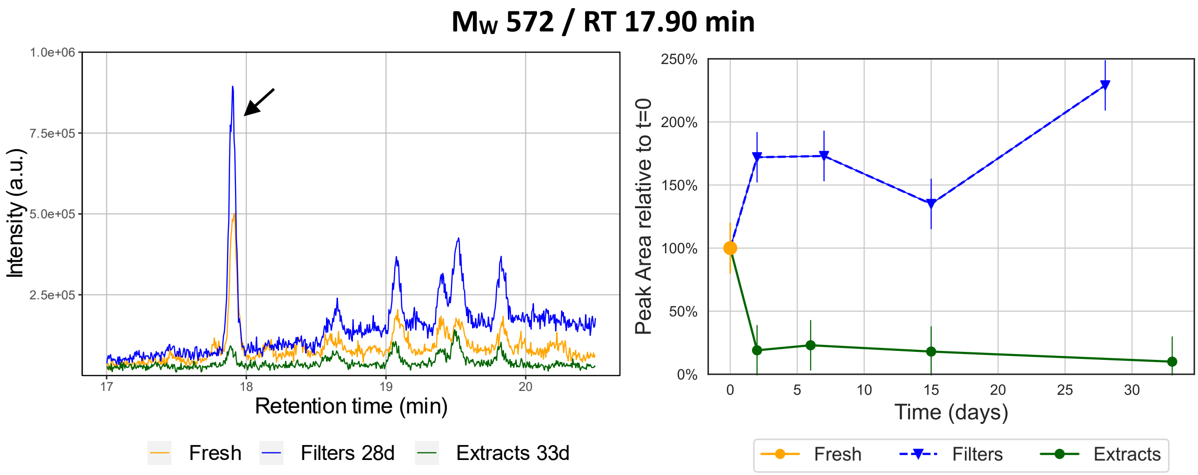

Figure S 25: EIC of the m/z 571.3123 compound tentatively assigned as a trimer. The corresponding timeseries of the isomer is given on the right.

"Spiking" experiments

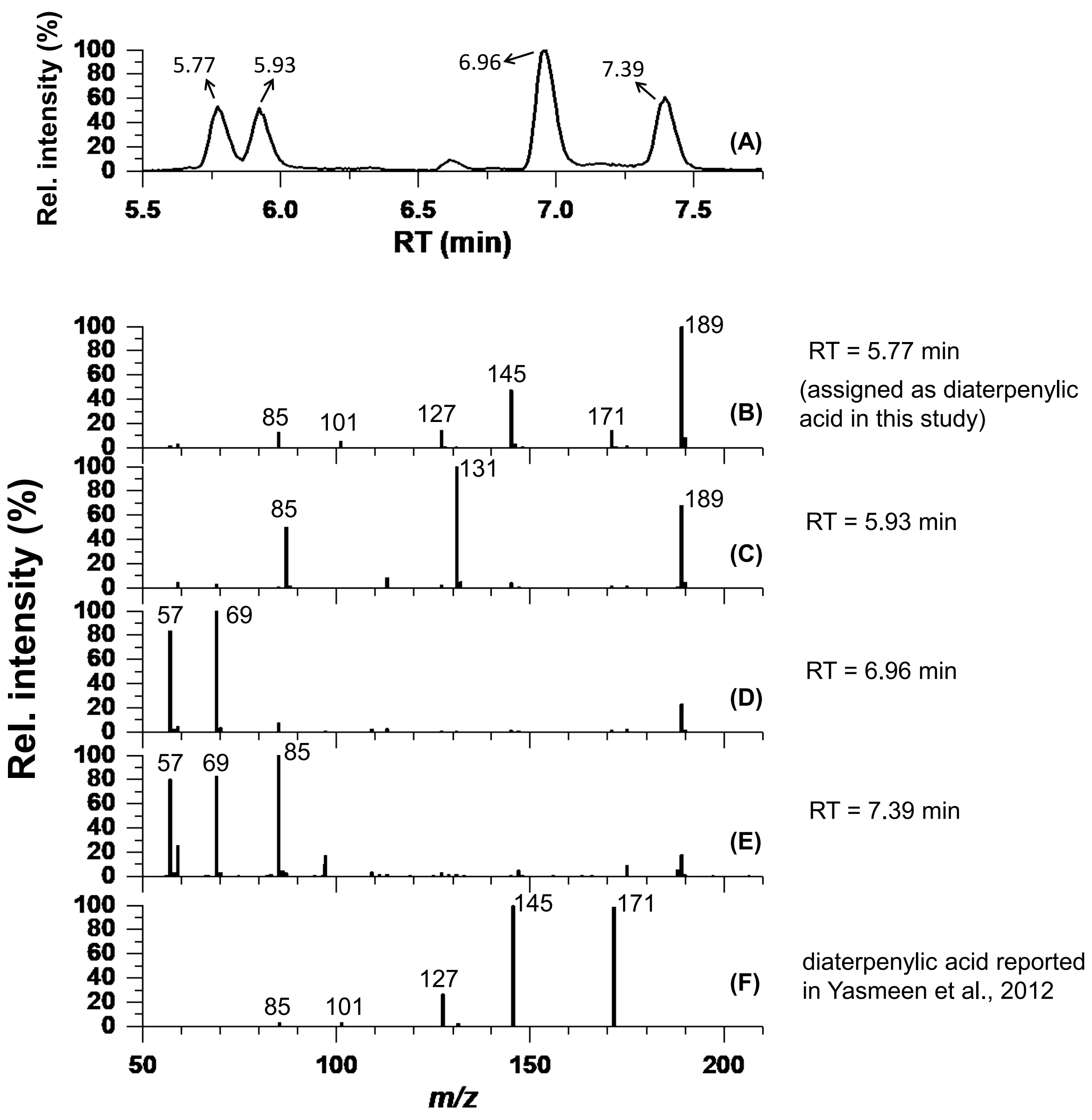

Figure S 26: EIC of m/z 189.0768 and the corresponding MS/MS results for each of the eluting isomer peaks at a set higher-energy collisional dissociation (HCD) of 10. The isomer eluting at 5.77 min shows the same fragmentation pattern as diaterpenylic acid in Yasmeen et al., (2012)[9]. (F) reconstructed from data in Yasmeen et al., (2012)[9].

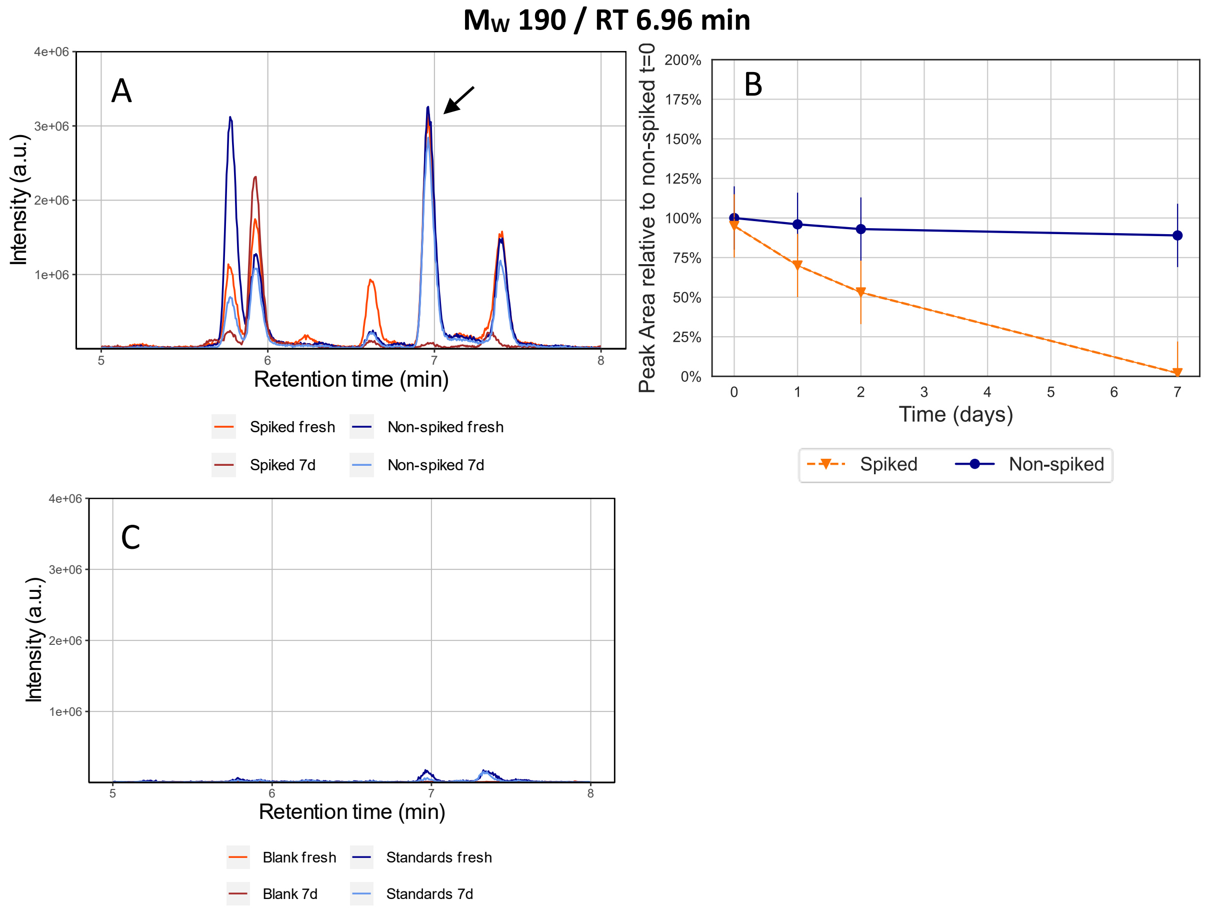

Figure S 27: (A) EIC of an isomer of diaterpenylic acid with m/z 189.0776 eluting at 6.96 min in the "non-spiked" and "spiked" fresh and 7-day old filter samples. (B) The timeseries of this isomer is given on the right. (C) EICs of fresh and stored blank and “standards only” samples of m/z 189.0776.

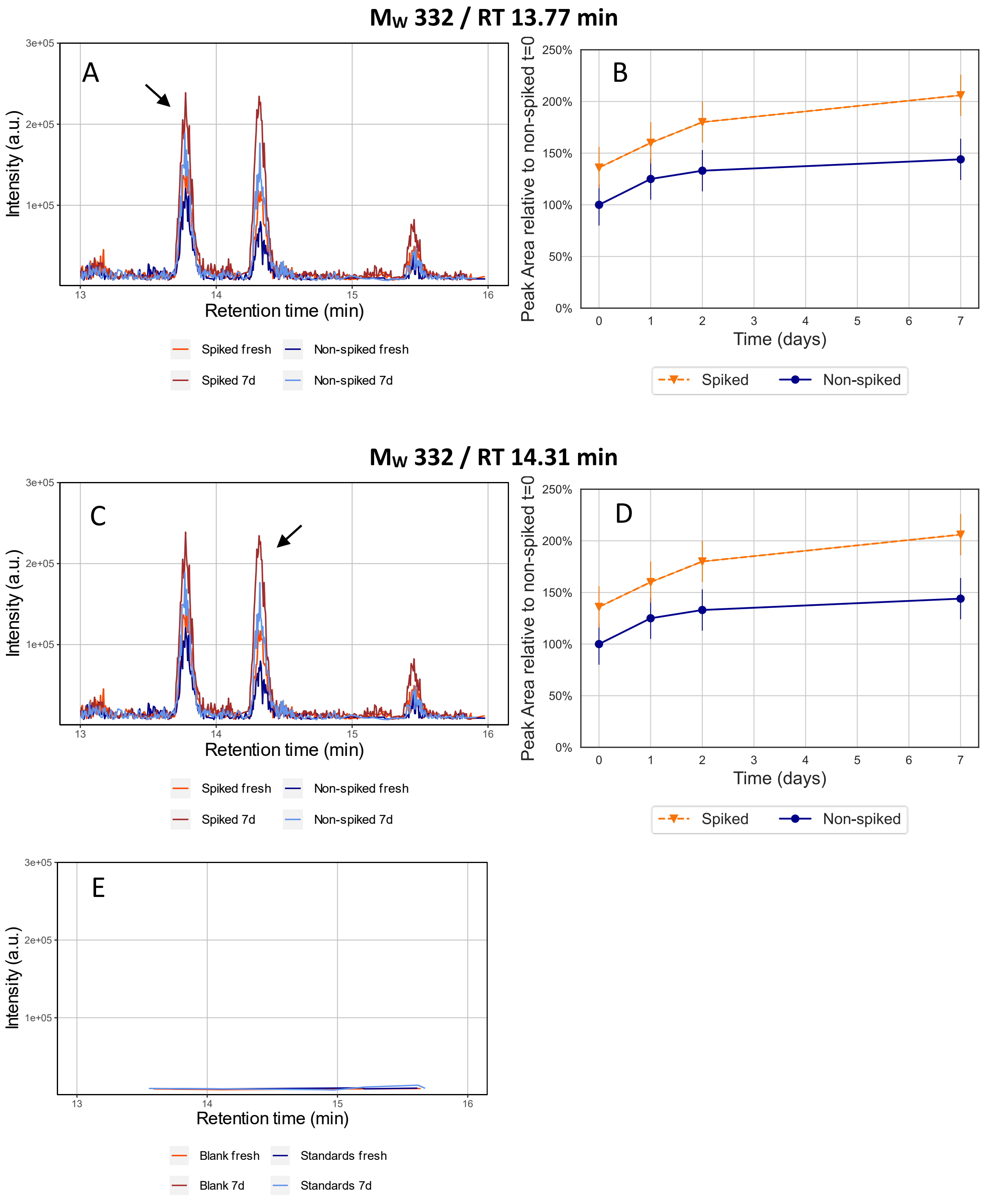

Figure S 28: (A,C) EICs of isomers of the m/z 331.1393 proposed to be an ester of diaterpenylic acid and pimelic acid eluting at 13.77 and 14.31 min in the "non-spiked" and "spiked" fresh and 7-day old filter samples. (B,D) The timeseries of these isomers are given on the right. (E) EICs of fresh and stored blanks and “standards only” samples of m/z 331.1393.

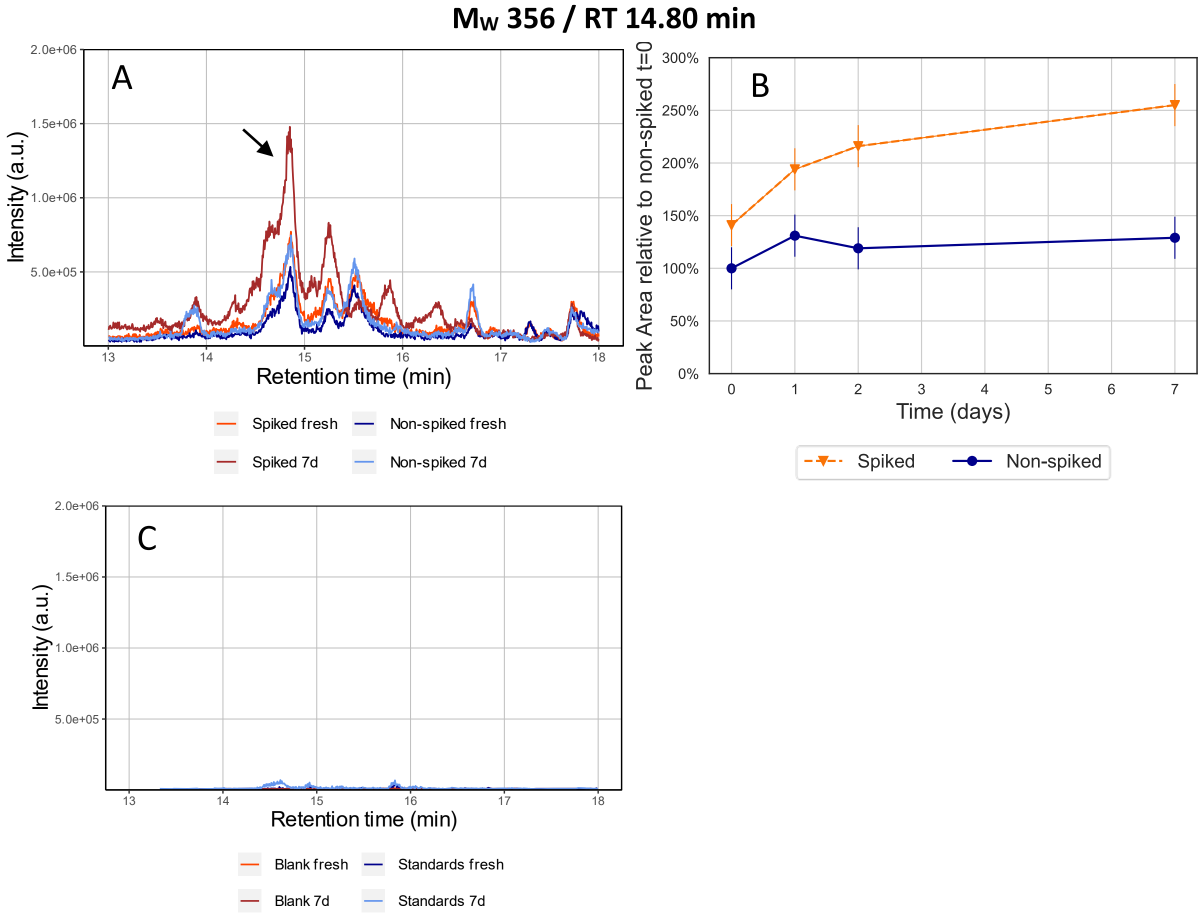

Figure S 29: (A) EIC of an isomer of the m/z 355.1757 proposed to be an ester of diaterpenylic acid and cis-pinonic acid eluting at 14.80 min in the "non-spiked" and "spiked" fresh and 7-day old filter samples. (B) The timeseries of this isomer is given on the right. (C) EIC of fresh and stored blank and “standards only” samples of m/z 355.1757.

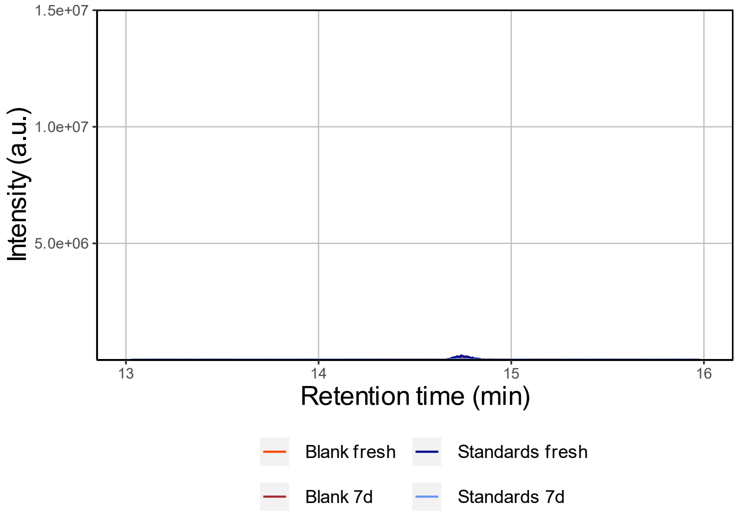

Figure S 30: EIC of fresh and aged blank and “standards only” samples of m/z 357.1550. The corresponding EICs are given in the main text Figure 5.

References

(1) Resch, J.; Wolfer, K.; Barth, A.; Kalberer, M. Effects of Storage Conditions on the Molecular-Level Composition of Organic Aerosol Particles. *Atmos. Chem. Phys.* **2023**, *23* (16), 9161–9171. https://doi.org/10.5194/acp-23-9161-2023.

(2) Kristensen, K.; Watne, Å. K.; Hammes, J.; Lutz, A.; Petäjä, T.; Hallquist, M.; Bilde, M.; Glasius, M. High-Molecular Weight Dimer Esters Are Major Products in Aerosols from α-Pinene Ozonolysis and the Boreal Forest. *Environ. Sci. Technol. Lett.* **2016**, *3* (8), 280–285. https://doi.org/10.1021/acs.estlett.6b00152.

(3) Kenseth, C. M.; Huang, Y.; Zhao, R.; Dalleska, N. F.; Caleb Hethcox, J.; Stoltz, B. M.; Seinfeld, J. H. Synergistic O 3 + OH Oxidation Pathway to Extremely Low-Volatility Dimers Revealed in β-Pinene Secondary Organic Aerosol. *Proc. Natl. Acad. Sci. U. S. A.* **2018**, *115* (33), 8301–8306. https://doi.org/10.1073/pnas.1804671115.

(4) Kourtchev, I.; Doussin, J. F.; Giorio, C.; Mahon, B.; Wilson, E. M.; Maurin, N.; Pangui, E.; Venables, D. S.; Wenger, J. C.; Kalberer, M. Molecular Composition of Fresh and Aged Secondary Organic Aerosol from a Mixture of Biogenic Volatile Compounds: A High-Resolution Mass Spectrometry Study. *Atmos. Chem. Phys.* **2015**, *15* (10), 5683–5695. https://doi.org/10.5194/acp-15-5683-2015.

(5) Kristensen, K.; Cui, T.; Zhang, H.; Gold, A.; Glasius, M.; Surratt, J. D. Dimers in α-Pinene Secondary Organic Aerosol: Effect of Hydroxyl Radical, Ozone, Relative Humidity and Aerosol Acidity. *Atmos. Chem. Phys.* **2014**, *14* (8), 4201–4218. https://doi.org/10.5194/acp-14-4201-2014.

(6) Yasmeen, F.; Vermeylen, R.; Szmigielski, R.; Iinuma, Y.; Böge, O.; Herrmann, H.; Maenhaut, W.; Claeys, M. Terpenylic Acid and Related Compounds: Precursors for Dimers in Secondary Organic Aerosol from the Ozonolysis of α- and β-Pinene. *Atmos. Chem. Phys.* **2010**, *10* (19), 9383–9392. https://doi.org/10.5194/acp-10-9383-2010.

(7) Sato, K.; Jia, T.; Tanabe, K.; Morino, Y.; Kajii, Y.; Imamura, T. Terpenylic Acid and Nine-Carbon Multifunctional Compounds Formed during the Aging of β-Pinene Ozonolysis Secondary Organic Aerosol. *Atmos. Environ.* **2016**, *130*, 127–135. https://doi.org/10.1016/j.atmosenv.2015.08.047.

(8) Müller, L.; Reinnig, M. C.; Warnke, J.; Hoffmann, T. Unambiguous Identification of Esters as Oligomers in Secondary Organic Aerosol Formed from Cyclohexene and Cyclohexene/α-Pinene Ozonolysis. *Atmos. Chem. Phys.* **2008**, *8* (5), 1423–1433. https://doi.org/10.5194/acp-8-1423-2008.

(9) Yasmeen, F.; Vermeylen, R.; Maurin, N.; Perraudin, E.; Doussin, J. F.; Claeys, M. Characterisation of Tracers for Aging of α-Pinene Secondary Organic Aerosol Using Liquid Chromatography/Negative Ion Electrospray Ionisation Mass Spectrometry. *Environ. Chem.* **2012**, *9* (3), 236–246. https://doi.org/10.1071/EN11148.
